# ATP-Binding Cassette (ABC) Transporter Genes Involved in Pyrethroid Resistance in the Malaria Vector *Anopheles sinensis*: Genome-Wide Identification, Characteristics, Phylogenetics, and Expression Profile

**DOI:** 10.3390/ijms20061409

**Published:** 2019-03-20

**Authors:** Qiyi He, Zhentian Yan, Fengling Si, Yong Zhou, Wenbo Fu, Bin Chen

**Affiliations:** 1 School of Life Sciences, Chongqing University, Chongqing 401331, China; hqy7171@126.com; 2 Chongqing Key Laboratory of Vector Insects, Institute of Entomology and Molecular Biology, Chongqing Normal University, Chongqing 401331, China; yzt318@163.com (Z.Y.); sifengling1217@163.com (F.S.); zhouyong_320@163.com (Y.Z.); fuice@126.com (W.F.)

**Keywords:** *Anopheles sinensis*, ATP-Binding Cassette (ABC) transporters, pyrethroid resistance, deltamethrin, gene expression, RNAseq analysis

## Abstract

**background**: The ATP-binding cassette (ABC) transporters family is one of the largest families of membrane proteins existing in all living organisms. Pyrethroid resistance has become the largest unique obstacle for mosquito control worldwide. ABC transporters are thought to be associated with pyrethroid resistance in some agricultural pests, but little information is known for mosquitoes. Herein, we investigated the diversity, location, characteristics, phylogenetics, and evolution of ABC transporter family of genes in the *Anopheles sinensis* genome, and identified the ABC transporter genes associated with pyrethroid resistance through expression profiles using RNA-seq and qPCR. Results: 61 ABC transporter genes are identified and divided into eight subfamilies (ABCA-H), located on 22 different scaffolds. Phylogenetic and evolution analyses with ABC transporters of *A. gambiae*, *Drosophila melanogaster*, and *Homo sapiens* suggest that the ABCD, ABCG, and ABCH subfamilies are monophyly, and that the ABCC and ABCG subfamilies have experienced a gene duplication event. Both RNA-seq and qPCR analyses show that the *AsABCG28* gene is uniquely significantly upregulated gene in all three field pyrethroid-resistant populations (Anhui, Chongqing, and Yunnan provinces) in comparison with a laboratory-susceptible strain from Jiangsu province. The *AsABCG28* is significantly upregulated at 12-h and 24-h after deltamethrin exposure in three-day-old female adults. Conclusion: This study provides the information frame for ABC transporter subfamily of genes, and lays an important basis for the better understanding and further research of ABC transporter function in insecticide toxification. The *AsABCG28* gene is associated with pyrethroid detoxification, and it functions at later period in the detoxification process for xenobiotics transportation.

## 1. Introduction

ATP-binding cassette (ABC) transporters constitute one of the largest families of membrane proteins, and have been found in all living organisms from bacteria to human being. The majority of these proteins function as the ATP-dependent transport of various substrates (e.g., sugars, amino acids, lipids, peptides, vitamins, sterols, hormones, metal ions, xenobiotics, and chemotherapeutic drugs) across lipid membranes [1,2]. ABC transporters share highly conserved domains with all members containing the highly conserved cytosolic nucleotide-binding domains (NBDs, also called ATP-binding cassettes), and most members also containing less conserved transmembrane domains (TMDs) [3]. The NBDs contain several conserved motif sequences, such as ABC-signature (LSGGQ), Walker A-motif, Walker B-motif, Q-loop, and H-motif, which bind and hydrolyze ATP to energize the transport cycle. The TMDs usually consist of 5–6 membrane-spanning helices that provide substrate specificity and translocation [4]. The ABC signature motif can be uniquely used to identify ABC transporters [5]. In eukaryotes, ABC transporters are either full transporters containing four domains (two NBDs and two TMDs) in one polypeptide, or half-transporters only containing two domains (one TMD and one NBD), and the later need to homo- or heterodimerize to form a functional ABC transporter with at least four domains: two NBDs and two TMDs [6]. Based on the sequence similarity of the NBDs, the ABC transporter family of genes is classified into eight subfamilies termed from ABCA to ABCH [1]. Up to now, the ABC transporter genes have been identified in some insect species at genome-wide level, including *Drosophila melanogaster* (56 ABC transporter genes) [1], *Bombyx mori* (51) [7], *Apis mellifera* (41) [4,7], *Anopheles gambiae* (52) [7], and *Tribolium castaneum* (73) [8].

Many mosquito species are main disease vectors, and chemical control with insecticides is still the most critical measure in the mosquito control program. The pyrethroid is the only insecticide recommended by the World Health Organization (WHO) in the program, and is widely used for the treatment of mosquito net and indoor residual spraying [9]. However, the significant increasing of pyrethroid resistance has become the unique, largest obstacle for the mosquito control worldwide [10,11,12]. The molecular mechanisms responsible for pyrethroid resistance are complicated, including the alteration of the target sites in the para-type sodium channel, which leads knockdown resistance, and the over expression of detoxification enzymes, such as P450s, esterases, and GSTs, which leads metabolic resistance [13,14,15]. In recent years, the role of ABC transporters has also been reported to be associated with pyrethroid resistance in some species of agricultural and medical importance [4]. In mosquitoes, microarray experiments revealed that four ABC transporter genes were significantly upregulated in a pyrethroid-resistant strain of the dengue vector *Aedes aegypti*. Quantitative PCR showed that the *ABCB4* gene was upregulated approximately seven times in an *A. aegypti*-resistant strain compared to the susceptible strain [16]. RNA-seq analysis revealed that the *AGAPOO8436* gene (ABCC subfamily) was significantly upregulated in a deltamenthrin-resistant strain of *A. gambiae* [17]. More recently, the expression profile analysis of ABC transporter genes in permethrin-treated larvae and adults of *A. stephensi* showed that *AnstABCBmember6* and *AnstABCG4* were significantly upregulated, which indicates that these ABC transporters might be relevant to insecticide defense [18,19,20]. However, the expression profile of ABC transporter genes involved in pyrethroid resistance has never been investigated in different field populations with pyrethroid resistance in a given species.

The mosquito species *Anopheles sinensis* is a major malaria vector in China and other Southeast Asian countries [21]. Due to extensive and continued application of pyrethroid insecticides, pyrethroid resistance is now widespread in *A. sinensis* in China as well as South Korea [22]. We have deeply sequenced and assembled the genome of *A. sinensis* [15,21,23], and we are conducting comprehensive research on the molecular mechanism of pyrethroid resistance using the species as model. Some families of genes have been identified at whole-genome level and their association with pyrethroid resistance have been investigated or summarized, such as CCEs [14], P450s [15], CSPs [23], CPs [24], OBPs [25], and iGluRs [26]. However, little information has been known about the ABC transporter genes and their association with pyrethroid resistance in *A. sinensis*. In the present study, we identified and classified the ABC transporter genes at whole-genome level in *A. sinensis*, and characterized these ABC transporters, including motif pattern, genomic distribution, gene structure, and phylogenetic relationship. More importantly, we compared the expression profiles of these ABC transporter genes from *A. sinensis* pyrethroid-resistant field populations and pyrethroid-susceptible laboratory strain by RNA-seq and RT-qPCR, analyzed the timescale transcriptional response of representative ABC transporter genes after pyrethroid treatment of pyrethroid-resistant laboratory strain, and identified and discussed the ABC transporter genes associated with pyrethroid resistance. The study confirms that ABC transporters play an important role in pyrethroid resistance, and provides the necessary basis to further elucidate the mechanism of ABC transporters in pyrethroid resistance in *A. sinensis*.

## 2. Results and Discussion

### 2.1. Diversity and Genomic Location of the A. sinensis ABC Transporter Genes

A total of 61 putative ABC transporter genes are identified from *A. sinensis* genome. Of them, 58 genes have evidences of mRNA expression with EST sequences, and other three genes all have full-length protein-coding sequences extracted from the genome, although they do not have expression sequence support. The nomenclature of all these genes is in reference of that of *A. gambiae* [27], as well as the motif and phylogenetic analyses in the present study. *AsABCA8* is the longest ABC transporter gene—encoding 1993 amino acids (a.a.)—and *AsABCG24* is the shortest one, encoding 528 a.a. The information of genome position, exon number, gene length, amino acid size, and EST of the 61 ABC transporter genes identified are summarized in Table 1. The ABC transporter number of *A. sinensis* is similar to that in the *D. melanogaster* genome (56 ABC transporter genes)^1^, but more than that in *A. mellifera* (41) [4,7], *B. Mori* (51) [7], and *A. gambiae* genomes (52) [7], and less than that in *T. castaneum* (73) [8] (Table 2).

**Figure 1:**
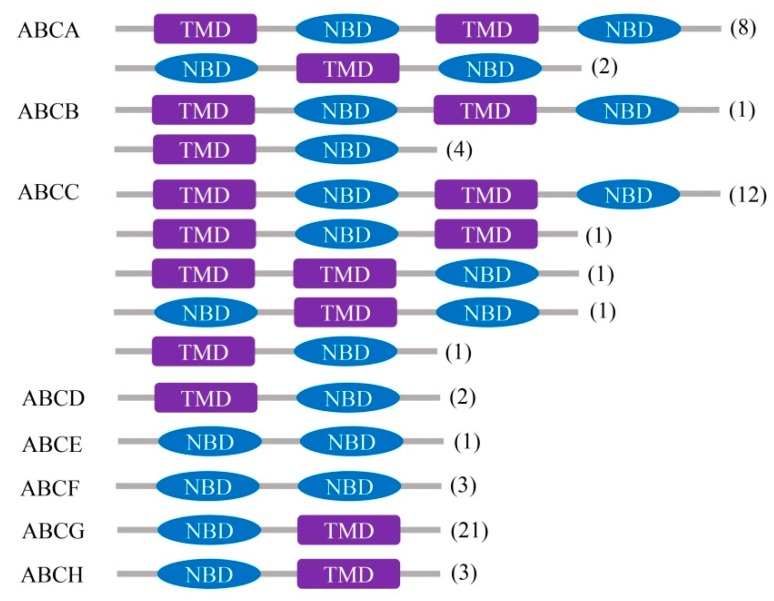
Conserved domains of *A. sinensis* ABC transporters. Gray bars represent the amino acid sequences of each structure type of ABC transporter genes. The subfamily names of the ABC transporters are marked on the left side of the structure types, and the gene numbers of each structure type are denoted in brackes on the right side of the gray bars. The purple-filled rectangular boxes denote transmembrane domain (TMD) positions on the amino acid sequences, and the blue-filled oval boxes indicate nucleotide binding domain (NBD) positions. The TMD and NBD domains of each *A. sinensis* ABC transporter were identified using the program Pfam.

All 61 *A. sinensis* ABC transporter genes possess one or two conserved and characteristic NBDs (Figure 1). The ABCA, ABCB, and ABCC subfamilies contain eight, one, and 12 full transporter genes (two NBDs and two TMDs), whereas the ABCD, ABCE, ABCF, ABCG, and ABCH subfamilies have no full transporter genes. The ABCA has two additional genes with two NBDs and one TMD, ABCB has four additional half-transporter genes with one NBD and one TMD, and ABCC has two additional genes with one NBD and two TMDs, one additional gene with two NBDs, and one TMD, and one half-transporter gene, respectively. The ABCD, ABCG, and ABCH subfamilies have two, 21, and three genes with all of them being half-transporter; however, the ABCE and ABCF subfamilies have one and three genes, respectively, all of them only contain two NBDs (without half-transporter).

**Figure 2:**
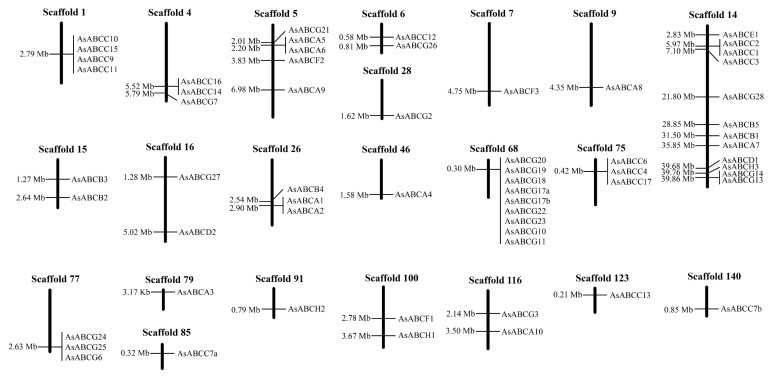
Distribution of *A. sinensis* ABC transporter genes on scaffolds. The number of left side on scaffold represents physical locations, and on the right side are gene names.

Genome mapping reveals that the 61 genes are located on 22 different scaffolds (Figure 2, Table 1). Among them, 19 genes (31.1% of all ABC transporter genes) are tandemly arranged into four clusters with at least three genes, 10 (16.4%) into five clusters with two genes, and 32 (52.5%) are present as singletons. For ABCC subfamily, four genes (4/16, 25.0%) were clustered on scaffold 1, three (3/16, 18.8%) on scaffold 75, two on scaffold 4, and two on scaffold 14; the other five genes are singly distributed. For ABCG subfamily, nine genes (9/21, 42.9%) are clustered on scaffold 68, three (3/21, 14.3%) on scaffold 77, two (2/21, 9.5%) on scaffold 14, and the remaining seven genes are singletons. For ABCA subfamily, there are four genes in two clusters belonging to scaffold 5 and scaffold 26, respectively, and the remaining six genes are singly distributed. The structure analysis shows that the exon number of the 61 *A. sinensis* ABC transporter genes is high complication ranging from two to 18 (Table 1). The majority of them have seven exons (27.9%, 17/61), followed by six (13.1%, 8/61), four (9.8%, 6/61), and five (9.8%, 6/61). In addition, nine genes have more than ten exons (14.8%, 10/61) and 12 genes have less than five exons (19.7%, 12/61). A total of 360 introns are predicted in all *A. sinensis* ABC transporter genes, whose sizes range from 13 to 9820 bp (444 bp in average), but the majority of them (64.2%, 231/360) displays sizes from 60 to 98 bp (Figure S1).

### 2.2. Phylogenetics and Characteristics of the A. sinensis ABC Transporter Genes

**Figure 3:**
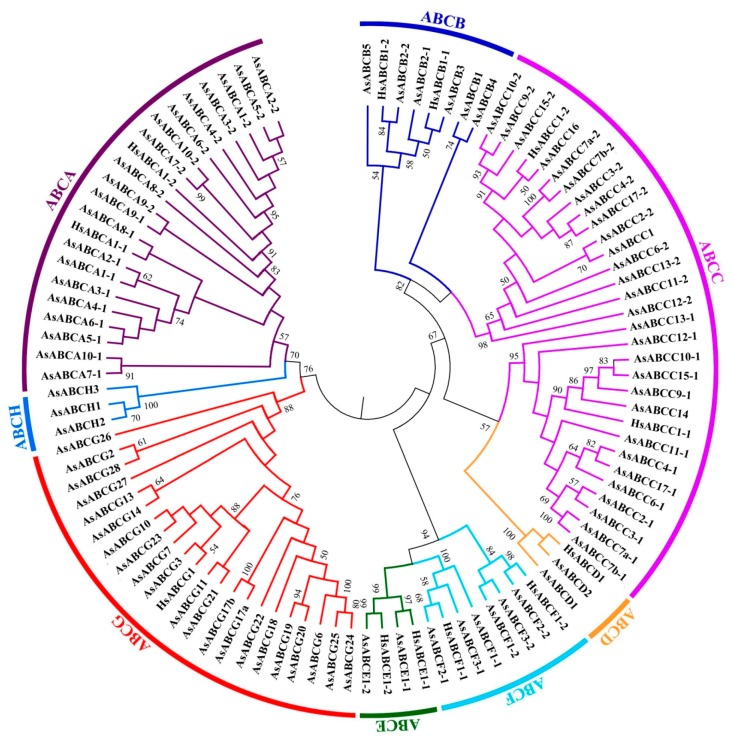
Phylogenetic relationships of the *A. sinensis* ABC transporter genes. The relationships were inferred based on the amino acid sequences of nucleotide binding domains (NBD) with maximum likelihood method using MEGA5.0, and those genes with two NBDs were treated as two separate gene taxa with each taxon using one different NBD amino acid sequence. The bootstrap values calculated from 1000 replicates were marked on each corresponding node. ABCA, ABCB, ABCC, ABCD, ABCE, ABCF, ABCG, and ABCH represent eight different subfamilies in the ABC transporter family.

Phylogenetic analysis reveals that the *A. sinensis* ABC transporter genes can be classified into eight subfamilies (A–H) with high bootstrap values at most of the respective brancges (Figure 3). The ABCD, ABCG, and ABCH subfamilies have two, 21, and three genes, respectively, and their branches have 100%, 88%, and 100% bootstrap values of support, respectively. These suggest that these three subfamilies are monophyly. The ABCA subfamily has only 57% bootstrap value of support, and it appears to be closer in phylogenetics with ABCH, both together having a 70% bootstrap value. The ABCB, ABCC, and ABCF subfamilies appear to be paraphyly with ABCB, having closer relationship with ABCC, ABCC with ABCB and ABCD, and ABCF with ABCE. We separately investigated phylogenetic relationships of ABC transporter genes in each subfamily with inclusion of the ABC transporter sequences of *A. gambiae*, *D. melanogaster*, and *H. sapiens* to further understand the evolution and characteristics.

ABCA subfamily (Figure S2A). Ten ABCA subfamily genes were identified in the *A. sinensis* genome. Phylogenetic analysis reveals that the *A. gambiae* ABCA genes each have one ortholog with that of *A. sinensis*, except for the AGAP001523 that is sister with *AsABCA7* + *AsABCA10*. These three genes (their clade with 100% bootstrap value support) form a sister group with *DmCG31731* that is involved in programmed cell death in *D. melanogaster* metamorphosis [28]. In *A. sinensis*, the ABCA subfamily contains eight full transporters (2TMDs and 2NBDs) and two incomplete ABC transporters (1TMD and 2NBDs). Interestingly, all nine ABCA transporters in *A. gambiae* are full transporters [7], the same as the water flea, *Daphnia pulex* [29]. However, both full and half-transporters present in the ABCA subfamily of other insect species, such as *D. melanogaster*, *T. castaneum*, *A. mellifera*, and *B. Mori* [7]. All ABCA transporters are full transporters in human [1], but no ABCA transporter has been found in the yeast *Saccharomyces cerecisiae* [30]. In the plant *Arabidopsis thaliana*, the ABCA subfamily consists of 11 half-transporters and only one full transporter [31]. Thus, the ABCA subfamily shows great variation among species.

ABCB subfamily (Figure S2B). In *A. sinensis*, five ABCB subfamily genes were identified, and each has distinct clades according to phylogenetic analysis. The *A. sinensis* ABCB subfamily consists of one full transporter and four half-transporters, which is consistent with *A. gambiae* and *A. mellifera* [4]. ABCB1 (MDR1/P-glycoprotein) was the first characterized human ABC transporter associated with multidrug resistance (MDR) phenotype to cancer, and later studies revealed additional MDRs in the ABCB subfamily [32]. In *D. melanogaster*, three ABCB full transporters were found and named Mdr49, Mdr50, and Mdr65. Mdr65 is involved in chemical protection of fruit fly brain by creating a blood–brain barrier (BBB) and Mdr49 performs a critical role in germ cell migration, while no further information is available on Mdr50 [33,34]. Furthermore, it was demonsterated that colchicine exposure and heat shock increased the expression of Mdr49 in *D. melanogaster* larvae, and exposure of *D. melanogaster* flies to polycyclic aromatic hydrocarbons led to an induced Mdr49 expression, suggesting that Mdr49 is involved in resistance to insecticides and other chemical compounds [35]. As shown in Figure S2B, *AsABCB2*, a full transporter, is clearly clustered with *A. gambiae AGAP005639* and *D. melanogaster DmMdr49* in phylogenetic analysis (bootstrap value of 100%). Particularly, *D. melanogaster* and other arthropod ABCB FT/P-gp have also frequently been linked to insecticide transporter and/or resistance [4,36], whether *AsABCB2* is involved in xenobiotic resistance requires further investigation.

ABCC subfamily (Figure S2C). In *A. sinensis*, there are 16 ABCC subfamily genes including 12 full transporters (two TMDs and two NBDs), 1 half-transporter (one TMD and one NBD), and three short form transporters (NBD-TMD-NBD, TMD-NBD-TMD, and TMD-TMD-NBD). In *D. melanogaster*, all 14 ABCC transporters are full transporters, whereas other insects such as *A. gambiae, Ap. mellifeta, B. Mori* and *T. castaneum* consist of different numbers of full, half, and incomplete ABCC transporters [7], implying that the ABCC subfamily diversely evolves in insects. In our phylogenetic analysis, two ABCCs (*AsABCC7a* and *AsABCC7b*) cluster together with high bootstrap support. Both genes show 99.72% identity in their amino acid sequences and have identical exon–intron structure (Figure S1), indicating they might have arisen by recent duplication events. *AsABCC12* clusters with *A. gambiae AGAP007917*, which is a putative ortholog of *D. melanogaster CG7806* and *HsABCC10/MRP7*. *HsABCC10/MRP7* has been porved to confer a *HsABCC1/MRP1*-type multidrug resistance phenotype in cellular models [37]. According to the phylogenetic analysis, *AsABCC6* and *AsABCC17* group together with a cluster of *D. melanogaster* ABCC transporters CG10505. CG10505 is regulated by heavy metals via metal-responsive transcription factor 1, and has been reported to be involved in biochemical detoxification of zinc and copper [38]. In addition, *AsABCC11* clusters with *A. gambiae AGAP008436* (99% of bootstrap value)*,* which has been detected with overexpression in a deltamenthrin-resistant strain of *A. gambiae* [17]. Thus, the potential roles of mosquito ABCC subfamily in detoxification is an important subject that needs to be developed.

ABCD subfamily (Figure S2D). The ABCD subfamily members are involved in the import of fatty acids and/or fatty acy1-CoAs into peroxisome [39]. Up to now, all identified ABCD transporters are known to be half-transporter and assembled as homodimers to function as transporters at the cellular level. In *A. sinesis*, we identified two ABCD subfamily genes, similar to the other species including *A. gambiae*, *A. mellifera*, *B. Mori*, *D. melanogaster*, and *T. castaneum* [4]. Based on phylogenetic analysis, each As-ABCD gene shows a clear homologous relationship to those of *A. gambiae, D. melanogaster,* and *H. sapiens*, indicating that the ABCD subfamily is evolutionarily conserved.

ABCE and ABCF subfamilies (Figure S2E). The ABCE and ABCF subfamilies consist of a pair of linked NBDs, but lack TMDs [40]. Therefore, both subfamilies are not involved in transmembrane functions, but they play a fundamental role in biological processes. In human, ABCE1 was identified as an inhibitor of RNase L and functioned in viral infection, tumor cell proliferation, antiapoptosis, translation initiation, elongation, termination, and ribosome biosynthesis, while human ABCF protein only participate in gene regulation system and ribosome assembly [41,42]. In insects, little has been known about both subfamilies. Recently, the injection of dsRNA specific for *TcABCE-3A* or *TcABCF-2A* of *T. castaneum* resulted in a lethal phenotype with 100% mortality in penultimate larvae, suggesting that they are active in cell viability [8]. In *A. sinensis*, only one ABCE gene and three ABCF genes were identified, as shown in most eukaryotes [4]. Phylogenetic analysis shows that each gene of both subfamilies grouped a unique cluster together with those of *A. gambiae*, *D. melanogaster*, and *H. sapiens*, indicating that they are highly conserved during evolution and probably have analogous functions as their human orothologus.

ABCG subfamily (Figure S2F). The ABCG subfamily only consists of half-transporters in most metazoans species, while several additional ABCG full transporters (also called pleiotropic drug resistance proteins (PDRs)) have been found in plant and fungi [43,44]. Twenty-one ABCG genes are identified in *A. sinensis*, suggesting that ABCG is the largest ABC subfamily in this species. All *A. sinensis* ABCG genes are half-transporters that possess a typical reverse domain architecture (NBD-TMD), and need to dimerize to form a functional transporters. The phylogenetic analysis reveals that *AsABCG17a* and *AsABCG17b* are neighbouring genes with 100% of bootstrap support. Both genes exhibit 100% amino acid identity and show a head-to-tail orientation (Table 1), suggesting they are the result of a tandem duplication.

In insects, the *D. melanogaster* ABCG genes white, scarlet, and brown are well studied and characterized as pigment precursor transporters into pigment cells [45,46]. The role of the *T. castaneum* and *B. Mori* ortholog of *D. melanogaster* white has also been experiemtally validated in eye pigmentation [8,47]. Phylogenetic analysis reveals that *D. melanogaster* white clusters with *A. gambiae AGAP000553* and *A. sinensis AsABCG27*, *D. melanogaster* scarlet clusters with *A. gambiae AGAP000133* and *A. sinensis AsABCG28*, and *D. melanogaster* brown clusters with *A. gambiae AGAP007655* and *A. sinensis AsABCG26*. However, whether all these ABC genes in *A. sinensis* are involved in the transportation of pigment precursor requires further investigation.

Most evidence has approved that ABCG subfamily was also involved in pesticide transporter and/or resistance [4]. Pedra et al. found an ABCG-type transporter gene that was significantly overtranscribed in both DDT-resistant 91-R and Wisconsin strains compared to the susceptible Canton-S flies [48]. You et al. showed by RNA-seq that ABCG transporters were more highly expressed in a *Plute xylostella* chlorpyrifos-resistant strain compared to a susceptible strain [49]. Yang et al. revealed by microarray gene expression studies that an ABCG transporter gene was highly overexpressed in adult *Bemisia tabaci* flies of a thiametaoxam resistant strain [50,51]. Jones et al. determined that one ABCG gene, ABCG4 (*AGAP001333*), was overexpressed (2-fold) in an *A. arabiensis* DTT-resistant stain than the susceptible strain [52]. Recently, Epis et al. investigated the expression profile of ABC transporter genes in *A. stephensi*, the results showed that *AnstABCG4* (*AGAP001333*) gene was upregulated expression in permethrin-trated larvae and adult mosquitoes compared to those of nontreated mosquitoes [19]. In our ABCG subfamily, *A. sinensis AsABCG28* clusters with *A. gambiae AGAP001333* with a 99% bootstrap value, indicating that *AsABCG28* might function in the defense against insectcide.

ABCH subfamily (Figure S2G). The ABCH subfamily was first identified in *D. melanogaster* and found in arthropods and zebrafish [1,4,53]. The domain architecture of the ABCH and ABCG genes is similar. In *A. sinensis*, three ABCH genes are found and process a reverse domain organization (NBD-TMD). Phylogenetic analysis shows that the ABCH genes are originated from a commom ancestor among the three insects. However, their physiological functions were poorly understood. Silva et al. deterimented that a ABCH gene was over expressed in adult aphids *Myzus persicae* upon exposure to pirimicarb [54]. You et al. found that an ortholog of *D. melanogaster CG9990* was the most upregulated ABCH transporter in a chlorpyrifos-resistant strain of *Plutella xylostella* compared to the susceptible stain [35]. Based on the ABCH phylogenetic analysis, *AsABCH2* is well clustered with *D. melanogaster CG9990*, highlighting the potential role of ABCH subfamily member in detoxification.

### 2.3. Ka/Ks of the Ortholog Genes in A. sinensis and A. gambiae

To better understand whether ABC transporter genes in *A. sinensis* and *A. gambiae* were under different evolutionary constraints, the pairwise *Ka*/*Ks* for each orthologous group was calculated. The results show that the purify selection is the main selection pressure driving the diversities of ABC transporters (all *Ka*/*Ks*<<1).

### 2.4. RNA-seq Expression Profile and qPCR Verification of ABC Transporters Associated with Pyrethorid Resistance

**Figure 4:**
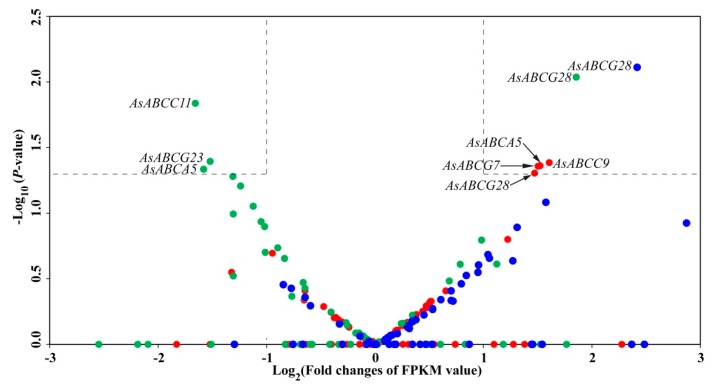
Expression profile of ABC transporter genes in three pyrethroid-resistent field populations (AH-FR, CQ-FR, and YN-FR) compared with a laboratory-susceptible strain (WX-LS), detected by RNA-seq. Genes with FPKM value ≥ 2 (i.e., ≥ 1 in Log_2_, showing on *X*-axis) and *p*-value < 0.05 (i.e., > 1.30 in –Log_10_, showing on *Y*-axis) are considered to be significantly upregulated, whereas those with FPKM value ≤ 0.5 (≤ −1 in Log_2_) and *p*-value < 0.05 are regarded as downregulated. Vertical dotted lines mark the 1 and −1 of Log_2_ (fold changes of FPKM value) values on the *X*-axis, and horizontal dotted lines denote the *p*-value = 0.05 of –Log_10_ (*p*-value) on the *Y*-axis. There are six genes that are significantly differently expressed in different populations, in which four genes are upregulated (*AsABCG28* commonly in three populations) and three genes are downregulated (AsABCA5 is upregulated in AH-FR but downregulated in CQ-FR). The red-, green-, and blue-filled circles indicate the AH-FR, CQ-FR, and YN-FR, respectively.

The expression profiles analysis of all ABC transporters from *A. sinensis* shows that six genes are significantly differentially expressed in at least one pyrethroid-resistant population (Figure 4, Table S1). *AsABCG28* is the uniquely significantly upregulated gene in the three resistant populations in comparison with susceptible WX-LS, with 2.69-fold upregulation (in AH-FR), 3.84-fold upregulation (CQ-FR), and 4.96-fold upregulation (YN-FR). The *AsABCC9*, *AsABCG7*, and *AsABCA5* are significantly upregulated in AH-FR by 2.96-fold, 2.75-fold, and 2.79-fold, respectively. Although the *AsABCA5* is also significantly upregulated in AH-FR, it is downregulated in CQ-FR. In contrast, *AsABCC11* and *AsABCG23* are significantly downregulated by 2.97-fold and 2.70-fold, respectively.

**Figure 5:**
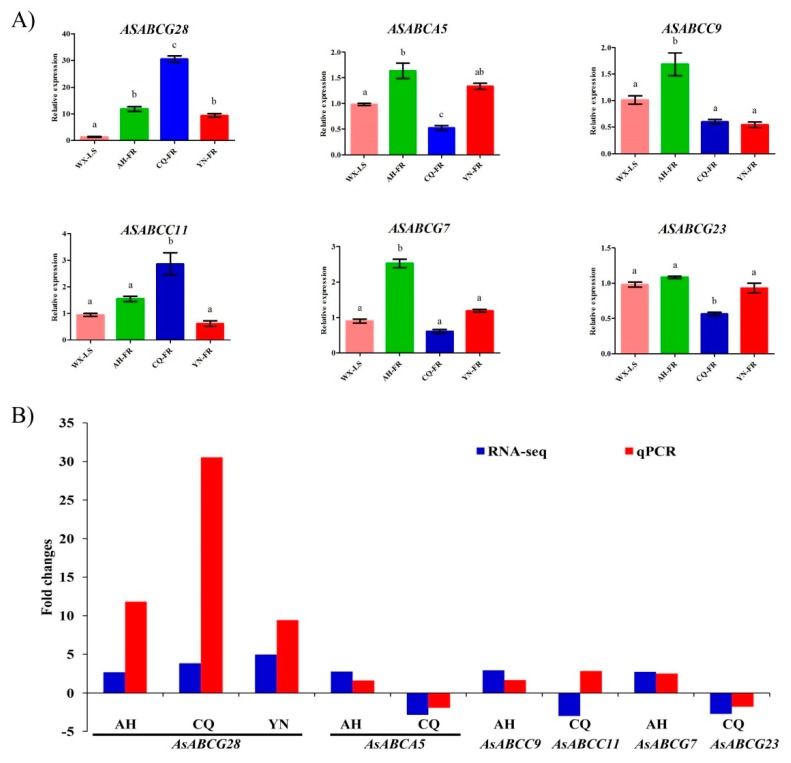
qPCR verification of six genes significantly differentially expressed in RNA-seq analysis. (**A**) The relative expression levels of these genes are shown as the mean ± SD of three biological replicates in qPCR analysis. The S7 (RPS7) and L49 (RPL49) genes were used as internal reference for expression normalization. The population/strain pairs marked with same letters are not significantly different in expression (*p*-value ≥ 0.05), and the remaining pairs are significantly different (*p*-value < 0.05), determined by one-way ANOVA. (**B**) Comparison of expression fold-changes between RNA-seq and qPCR analyses for the six genes in three field populations comparative with the laboratory-susceptible strain WX-LS. AH: Anhui population; CQ: Chongqing; YN: Yunnan.

In order to validate the results of the RNA-seq, these six genes with significant expression difference are selected for verification using qPCR analysis (Figure 5A) with gene-specific primers designed in the present study (Table 3). The results show that expression fold changes are basically consistent with those in RNA-seq analysis for five of these six genes tested (Figure 5B). The *AsABCG28* is also significantly upregulated in all three resistant populations in comparison to the susceptible strain WX-LS. The *AsABCG28* shares 76% a.a. identity with *AGAP001333* of *A. gambiae*, which has been reported to be significantly overexpressed in DDT resistance strain in *A. arabiensis* (as *ABCG4*) [52], and in thiamethoxam resistant strain in sweetpotato whitefly, *Bemisia tabaci* (as *ABCG4*) [55]. These findings imply that *AsABCG28* is an essential member involving defense against insecticide. *AsABCC9* and *AsABCG7* are significantly upregulated in AH-FR compared with WX-LS, which is the first report that these two genes are associated with pyrethroid resistance. *AsABCC11*, the homolog of *A. gambiae AGAP008436*, is significantly upexpressed in CQ-FR based on the qPCR analysis, consistent with earlier study that showes that *AGAP008436* is upregulated by 2.88-fold in detamethrin resistant strain in *A. gambiae* [17]. However, it is downexpressed in CQ-FR based on RNA-seq analysis. These results might countribute to the different batches of samples and/or the different splicing of the target gene. The *AsABCG23* is uniquely significantly downregulated in CQ-RF according to the qPCR analyses, consistent with the RNA-seq detection. The *AsABCA5* is significantly upexpressed in AH-FR compared with WX-LS, which agrees with an earlier study that shows that one ABCA gene is significantly upregulated in a chlorpyrifos-resistant strain of Lepidoptera *Pl. xylostella* [49]. However, the gene is downexpressed in CQ-FR, but no significant difference is observed in YN-FR. These findings indicated that the expression variation of *AsABCA5* might be due to the difference of geographical distribution in the mosquitoes investigated. Whether or not *AsABCA5* is related to pyrethroid resistance need to further investigate.

### 2.5. ABC Transporters Expression Subject to Deltamethrin Treatment

**Figure 6:**
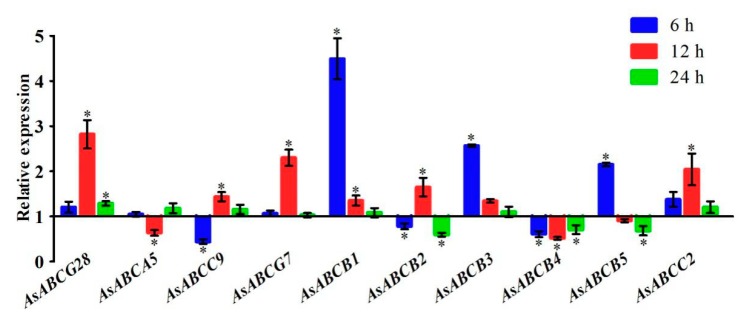
Expression changes of ten ABC transporter genes after pyrethroid treatment for *A. sinensis* female adults. The ten genes include four genes (*AsABCG28*, *AsABCA5*, *AsABCC9*, and *AsABCG7*) that are significantly upregulated in RNA-seq and qPCR analyses in different resistant population, and six genes (*AsABCB1*, *AsABCB2*, *AsABCB3*, *AsABCB4*, *AsABCB5*, and *AsABCC2*) that have been reported to be associated with pyrethroid resistance. The expression levels (means ± SD) were determined at 6-h, 12-h, and 24-h postpyrethroid treatment using qPCR with three biological replicates. The samples marked with “*” were significantly differently expressed (*p*-value < 0.05) compared with the untreated samples at the same time, and the samples without “*” were not significantly differently expressed.

A total of ten ABC transporter genes in *A. sinensis* (*AsABCG28*, *AsABCA5*, *AsABCC9*, *AsABCG7*, *AsABCB1*, *AsABCB2*, *AsABCB3*, *AsABCB4*, *AsABCB5*, and *AsABCC2*) were chosen to investigate the expression changes on timescale subject to deltamethrin treatment using three-day-old female adults of the laboratory deltamethrin-resistant strain WX-LR (Figure 6). The ten genes included four genes that showed significantly upexpression in RNA-seq analysis, and six genes that have been reported to be overexpressed in response to insecticide stress. The qPCR results show that three ABCB genes (*AsABCB1*, *AsABCB5*, and *AsABCB3*) are significantly overexpressed at 6-h after deltamethrin exposure. *AsABCB1* has the highest of overexpression (4.5-fold), and is also significantly upregulated in both larvae and adults of the mosquito *A. stephensi* after permethrin treatment (as *AnstABCBmember6*) [19,20]. The *AsABCB5* had 2.2-folds overexpression, and is also overexpressed in ivermectin-resistant *Rhipicephalus microplus* upon exposure to ivermectin (as *RmABCB10*) [56]. The *AsABCB3* had 2.6-fold overexpression, but no overexpression is observed in *A. stephensi* exposed to permethrin (as *AnstABCB3*) [19,20]. The detoxification process can be subdivided into four different phases (0–III), and involves a diversity of enzymatic complexes. ABC transporters are thought to act in phases 0 and III of the detoxification process [4]. In phase 0, ABC transporter genes belonging to the ABCB subfamily can directly extrude a wide range of xenobiotics out of cells, therefore avoiding the accumulation of these xenobiotics in the cells or organism [19]. The findings herein support the hypothesis that ABCB genes are involoved in early detoxification process after insecticide treatment.

Seven genes, inculding two ABCG genes (*AsABCG28* and *AsABCG7*), two ABCC genes (*AsABCC9* and *AsABCC2*), and three ABCB genes (*AsABCB1*, *AsABCB2*, and *AsABCB3*), show significantly upregulated expression 12-h after deltamethrin exposure (Figure 6). Most importantly, the *AsABCG28* has the highest overexpression (2.8 folds), and it is also significantly upregulated at 24-h. The *AnstABCG4*, the orthology of *AsABCG28* in *A. stephensi* showed similar expression pattern in permethrin-exposed larvae and adults [18,19,20]. Earlier reports showed that ABCG subfamily of genes participated in the transportion of xenobiotics modified by detoxifying enzymes in phase III [4,36]. Our research reveals that the *AsABCG28* is associated with pyrethroid detoxification, and it functions at later period in the detoxification process for xenobiotics transportion. In addition, the *AsABCC2* had 2.0-fold overexpression, and it is also significantly upregulated in pyrethroid-resistant strain of *A. aegypti* (as *ABCC2*) [16]. The *AsABCB2* had 1.6-fold overexpression at 12 h, but was downexpressed at 6-h and 24-h. This gene is significantly upregulated in *A. stephensi* at 1-h and 24-h after permethrin exposure (as *AnstABCB2*) [20], and in a susceptible strain of *A. aegypti* at 48-h post-temephos treatment (as *AaegP-gp*) [57]. A growing body of studies have indicated that the ABCB full transporter genes (P-gp) are associated with insecticide transport and/or resistance, including organophosphates [57], organochlorines [52], carbamates [58], and pyrethroids [59,60]. These results suggest that the *AsABCC2* and *AsABCB2* might participate in deltanethrin defense at the later phase of the detoxification.

The *AsABCB4* is significantly downregulated at all three time points investigated. However, microarray analysis showed that the *ABCB4* was significantly upregulated in different populations of DDT-resistant *A. gambiae* mosquitoes (as *AGP006364*) [61], and it was not differentially expressed or downregulated in both adults and larvae of *A. stephensi* (as *AnstABCB4*) [18,19,20]. The *AsABCB4* has a higher copy number in pyrethroid (permethrin and deltamethrin)-resistant *A. aegypti* populations compared to a laboratory-susceptible strain (as *ABCB4*) [16]. In addition, *AsABCB2* at 6 h and 24 h, *AsABCC9* at 6 h, *AsABCA5* at 12 h, and *AsABCB5* at 24 h herein also show significant downregulation. These findings could not reveal the regulation and function of the downregulation of these genes.

## 3. Materials and Methods

### 3.1. Mosquito Samples and Sequence Data

Two laboratory *A. sinensis* strains, WX-LS and WX-LR, susceptible and resistant to pyrethroid, respectively, were established in the Institute of Entomology and Molecular Biology, Chongqing Normal University, China. Field pyrethroid-resistant mosquitoes were collected from rice (*Oryza sativa*) fields in different locations of three geographical populations: Anhui (AH), Chongqing (CQ) and Yunnan (YN) provinces, China, between July and September, 2013.

*A. sinensis* genome data, including genome sequence and annotation, predicted gene set of sequences, and deduced amino acid sequences of the gene set, were obtained from the Institute of Entomology and Molecular Biology, Chongqing Normal University, China (publication in preparation). Two sets of transcriptome data were downloaded from the National Center for Biotechnology Information (NCBI) as an EST database (Accession numbers: SRA073189 and GAFE01000001-GAFE01028133). The ABC transporter amino acid sequences of *A. gambiae*, *D. melanogaster* and *Homo sapiens* were retrieved from NCBI as well (Table S2).

### 3.2. Identification and Characteristcs Analyses of A. sinensis ABC Transporter Genes

To identify putative ABC transporter genes, we firstly used the highly conserved NBD motif sequences of all ABC transporter genes from *D. melanogaster* and *A. gambiae* [7] as queries to conduct BLASTP searching against *A. sinensis* amino acid database with an e-value cut-off at 1e-6. Secondly, the Hidden Markov Model (HMM) for ABC transporter conservative motif ABC_tran (Pfam 00005) was downloaded from Pfam (http://pfam.xfam.org) and used as query to search the *A. sinensis* amino acid database using the program hmmsearch within the HMMER3 software Suite30, with an e-value threshold of 1e-10. Thirdly, all putative ABC transporter protein sequences obtained above were used as queries to perform TBLASTN searching against the *A. sinensis* genome with an e-value cut-off at 1e-5. Finally, the FGENSH+ (http://www.softberry.com) software was used to predict the genes with best matched individual ABC protein sequences in TBLASTN search results.

To further confirm the reliability of our results, all predicted ABC transporter genes were used as queries to perform BLASTN searches against the *A. sinensis* EST database [21,62]. A 95% or greater identity and minimum cut-off E-value (≤e-20) were employed to discriminate between duplicate genes. In addition, all genes were further analyzed by the program Pfam (http://pfam.xfam.org) to idientify its NBD and TMD domains. A BLASTN search using all obtained ABC sequences as quries was further conducted against the *A. sinensis* genome database to determine the location of ABC transporter genes on *A. sinensis* scaffolds. The structure of the ABC transporter genes were generated with GSDS (http://gsds.cbi.pku.edu.cn/) using intron/exon sites obtained from the *A. sinensis* genome database.

### 3.3. Phylogenetic Analysis of A. sinensis ABC Transporter Genes

The NBD sequences of preliminary gene models were extracted using the Prosite facility (http://prosite.expasy.org/prosite.html) with predicted protein sequences and the prosite profile PS50893. The NBD sequences were then aligned using ClustalW [63] and constructed phylogenetic relationships using maximum-likelihood method with Jones–Taylor–Thornton substitution model. The bootstrap values were calculated with 1000 replicates using the program package MEGA 5.0 [64]. Full-length sequences from *A. sinensis*, *A. gambiae*, *D. melanogaster*, and human ABC transporter protein sequences were used to carry out the phylogenetic analysis of each subfamily of ABC transporter genes using the same methodology as mentioned above.

### 3.4. Estimation of Synonymous Substitutions and Nonsynonymous Substitutions

The ABC transpoter sequences of each orthologs pair from *A. sinensis* and *A. gambiae* were first aligned using ClustalW software [63]. The files of the sequence alignments were then convented to PHYLIP alignment using MEGA 6. Finally, the convented files were uploaded to the YN00 program of PAML to estimate synonymous substitutions (*Ka*) and nonsynonymous substitution (*Ks*) [65]. *Ka/Ks* ratios of >1, <1, and =1 represent positive selection, stabilizing selection, and neutral selection, respectively.

### 3.5. RNA-seq Analysis and qPCR Verification of ABC Transporters Associated with Pyrethroid Resistance

The field-collected mosquito larvae and pupae were reared to adulthood at 27 ± 1 °C with 70 ± 10% relative humidity in local laboratories. The female adults of three days post emergence were identified to species morphologically, and then the *A. sinensis* mosquitoes were used to bioassay with 0.05% deltamethrin (Sigma-Aldrich, St. Louis, MO, USA) test papers according to the standard WHO tube bioassay [66]. More than 1000 mosquitoes per population collected from multiple sites in field were tested with 20 mosquitoes per tube, and paraffin oil-treated papers without deltamethrin were used as the control. After 60 min exposure to 0.05% deltamethrin, mosquitoes were transferred to recovery cups and maintained on a 6% sucrose solution for 24 h [15]. The mosquitoes alive 24 h after the recovery period were defined as resistant mosquitoes [66]. At least 100 resistant adult mosquitoes three days old from each population were preserved in RNAlater (Qiagen Shanghai, China) for RNA extraction and RNA-seq library construction, respectively. Three pyrethroid-resistant populations from Anhui (AH-FR), Chongqing (CQ-FR), and Yunnan (YN-FR) were used in the RNA-seq analysis and quantitative real-time Polymerase Chain Reaction (qPCR) verification, and the laboratory pyrethroid-susceptible strain (WX-LS) originally collected from Wuxi, Jiangsu province was handled with the same procedure.

Prior to RNA extraction, two or three legs of each individual mosquito were used to extract genomic DNA for molecular identification using the ribosomal DNA internal transcribed spacer rDNA-ITS2-based method [67]. Samples of 15 mosquitoes were pooled and prepared total RNA for each RNA-seq sample using Trizol Reagent (Invitrogen, Carlsbad, CA, USA) following the manufacturer protocol. Three biological replicates were set for three field-resistant populations and WX-LS strain. The quantity of total RNA was assessed using a NanoDrop spectrophotometer (NanoDrop Technologies, Thermo Fisher Scientific Inc., Waltham, MA, USA). RNA quality was assessed using an Agilent 2100 Bioanalyzer (Agilent, Santa Clara, CA, USA). The cDNA for each library was synthesized and amplified using the Mint-2 cDNA synthesis kit (Evrogen, Moscow, Russia). Illumina HiSeq™ 2000 was used for cDNA library sequencing at Beijing Genomics Institute (BGI), following the standard Illumina procedures.

TopHat was used to map reads obtained from Illumina sequencing to the *A. sinensis* genome [68]. Cufflinks was used to determined expressions in terms of fragments per kb per million reads (FPKM) [69]. Differential accumulation of transcripts between deltamethrin-resistant and -susceptible mosquitoes was assessed by the Cuffdiff program within Cufflinks. To minimize the impact of sequencing length and nucleotide composition, the FPKM for each gene of each sample was calculated to determine the expression level [70]. The normalized gene expression level of *A. sinensis* ABC transporter genes each in the three resistant populations was compared with the WX-LS strain. The genes that had the FPKM fold changes of ≥ 2 [log2 (fold change) ≥ 1] and the *p*-value ≤ 0.05 [−Log10 (*p*-value) ≥ 1.301] were considered as significantly upregulated between the resistant and susceptible strains. In contrast, the genes with the FPKM fold change ≤ 2 and the *p*-value ≤ 0.05 [−Log10 (*p*-value) ≥ 1.301] were regarded as significantly downregulated.

The ABC transporter genes to be significantly differentially expressed detected by RNA-seq analyses were chosen to perform qPCR. The expression confirmation of selected genes were investigated in the three deltamethrin resistant populations (AH-FR, CQ-FR, and YN-FR) using the laboratory-susceptible strain (WX-LS) as reference, and the qPCRs were carried out with three biological replicates (three mosquitoes per sample) and three technical replicates. Total RNA was extracted from four individual mosquitoes as the same conditions as RNA-seq with TRIzol Reagent (Invitrogen) following the manufacturer’s instructions. Complementary DNA was synthesized from 1.0 μg RNA using PrimScript TM RT Reagent Kit with gDNA Eraser (TaKaRa, Dalian, China) and stored at −20 °C. Real-time PCR was performed using CFX Connect Real-Time PCR System (Bio-Rad, USA). Each reaction with a final volumn of 15 μL was mixed with 7.5 μL of 2 x qPCR mix (Bio-Rad, Hercules, CA, USA), 0.5 μL each of gene-specific primers and 1 μL the cDNAs templates, and 5.5 μL of double distilled water. Running conditions were as follows: 94 °C for 3 min, 40 cycles of 95 °C for 5 s, 60 °C for 15 s, and 72 °C for 15 s. Gene-specific primes were designed using Primer Premier 5.0 [71]. The ribosomal protein S7 (RPS7) and the ribosomal protein L49 (RPL49) were used as internal controls. The relative expression levels of each gene in four populations/strain were normalized in comparison with RPS7 and RPL49 CT values using the 2^−ΔΔ*C*t^ method [72]. All data were shown as mean ± SD (standard deviation). The statistical significance of the gene expressions was calculated using one-way Analysis of Variance (ANOVA) and a value of *p* < 0.05 was considered statistically significant.

### 3.6. Expression Changes of Pyrethroid Resistance-Relative ABC Transporter Genes Subject to Deltamethrin Treatment

To analyse the expression profiles of ABC transporter genes after deltamenthrin treatment, the bioassays were conducted on three days old female mosquitoes following the standard WHO tube test [66]. Batches of 20–25 mosquitoes from *A. sinensis* strain WX-LR were exposed to test papers impregnated with 0.05% deltamethrin (Sigma-Aldrich, St. Louis, MO, USA) for one hour. After exposure, the mosquitoes were then transferred to recovery cups and supplied with 6% glucose solution. Tests with non-impregnated control papers were used as controls. Survivors and nonexposed mosquitoes were collected at different times (6-h, 12-h, and 24-h) postrecovery. The mosquitoes were stored in RNAlater (Qiagen Shanghai, China) and kept at −80 °C for molecular analysis.

Based on the RNA-seq and qPCR analyses in the present study and previously published literature [4,20], the expression of selective ABC transporter genes from *A. sinensis* were investigated at three time-points (6-h, 12-h, and 24-h) after recovery. The relative gene expressions of all selective genes were determined by qPCR as mentioned above. Three biological replicates were run for each sample on a plate. The expression levels of target genes were calculated relative to the internal reference genes. For each target gene, the expression level of the control group was considered as the basal level (set equal to 1). The relative expression level in the treated group was showed as means ± SD. The expression level between treated and control groups were calculated using a Student’s *t*-test and a value of *p* < 0.05 was considered statistically significant.

## 4. Conclusions

The study provides useful insights into the diversity, location, characteristics and phylogenetics of ABC transporter family of genes in *A. sinensis* genome, and ABC transporter genes associated with pyrethroid resistance. The ABCD, ABCG, and ABCH subfamilies are monophyly, and the ABCC and ABCG subfamilies experience gene duplication event. The *AsABCG28* gene is associated with pyrethroid detoxification, and it functions at later period in the detoxification process for xenobiotics transportion. This study provides the information frame for ABC transporter subfamily of genes, and lays an important basis for the better understanding and further research of ABC transporter function in insecticide toxification.

## Figures and Tables

**Table 1:** Information summary of 61 ABC transporter genes identified in the *A. sinensis* genome.

Gene Name	Gene Annotation ID	Genome Location ^a^	Number of Exon	Gene Length (bp) (Exon + Intron)	Amino Acid Number (AA)	EST Support ^b^
**ABCA Subfamily (10 genes)**						
*AsABCA1*	ASI10006665	scaffold26:2898415-2904542(−)	10	4839+1289	1613	+
*AsABCA2*	ASI10006666	scaffold26:2907038-2912368(−)	7	5304+3051	1768	+
*AsABCA3*	ASI10001787	scaffold79:3171-8550(+)	7	4956+424	1652	+
*AsABCA4*	ASI10005446	scaffold46:1576155-1586398(−)	7	4965+5279	1655	+
*AsABCA5*	ASI10011585	scaffold5:2204987-2211927(−)	8	4989+1952	1663	+
*AsABCA6*	ASI10011586	scaffold5:2214289-2220237(−)	6	4887+1062	1629	+
*AsABCA7*	ASI10017076	scaffold14:35853362-35861993(−)	7	4941+1825	1647	+
*AsABCA8*	ASI10008148	scaffold9:4353984-4361438(+)	9	5979+1476	1993	+
*AsABCA9*	ASI10012001	scaffold5:6985656-6991716(−)	14	5007+1054	1669	+
*AsABCA10*	ASI10008529	scaffold116:3498475-3502806(−)	5	3417+915	1139	+
**ABCB Subfamily (5 genes)**						
*AsABCB1*	ASI10016633	scaffold14:31503016-31505870(−)	4	2547+308	849	+
*AsABCB2*	ASI10010010	scaffold15:2635069-2641163(+)	11	3882+2213	1294	+
*AsABCB3*	ASI10009929	scaffold15:1271785-1274177(−)	4	2181+212	727	+
*AsABCB4*	ASI10006641	scaffold26:2544630-2550018(+)	7	2280+3109	760	+
*AsABCB5*	ASI10016463	scaffold14:28845118-28848788(−)	9	1965+1706	655	+
**ABCC Subfamily (16 genes)**						
*AsABCC1*	ASI10014633	scaffold14:5983931-5989564(+)	7	3534+2100	1178	+
*AsABCC2*	ASI10014632	scaffold14:5970014-5980211(−)	6	4140+6058	1380	+
*AsABCC3*	ASI10014742	scaffold14:7100932-7109199(+)	9	4347+3921	1449	+
*AsABCC4*	ASI10004082	scaffold75:426548-436333(−)	12	4404+5382	1468	+
*AsABCC6*	ASI10004081	scaffold75:419144-426092(−)	11	3972+2977	1324	-
*AsABCC7a*	ASI10001723	scaffold85:324079-329862(+)	7	4236+1548	1412	+
*AsABCC7b*	ASI10002723	scaffold140:852869-858944(+)	7	4236+1840	1412	+
*AsABCC9*	ASI10007114	scaffold1:2805165-2810461(−)	6	4512+785	1504	+
*AsABCC10*	ASI10007112	scaffold1:2792623-2797620(−)	7	4521+477	1507	+
*AsABCC11*	ASI10007115	scaffold1:2812936-2817655(−)	4	4212+508	1404	+
*AsABCC12*	ASI10003245	scaffold6:579715-584317(−)	3	4416+187	1472	+
*AsABCC13*	ASI10001014	scaffold123:207484-225544(−)	18	4017+14044	1339	+
*AsABCC14*	ASI10009681	scaffold4:5523940-5529838(−)	11	3453+2446	1151	+
*AsABCC15*	ASI10007113	scaffold1:2799112-2804048(−)	7	4515+422	1505	+
*AsABCC16*	ASI10009679	scaffold4:5516323-5523342(−)	9	2199+4821	733	+
*AsABCC17*	ASI10004083	scaffold75:439279-443721(−)	4	4203+240	1401	+
**ABCD Subfamily (2 genes)**						
*AsABCD1*	ASI10017457	scaffold14:39675247-39680568(−)	6	2226+3096	742	+
*AsABCD2*	ASI10013565	scaffold16:5022027-5030607(−)	8	2085+6496	695	+
**ABCE Subfamily (1 gene)**						
*AsABCE1*	ASI10014410	scaffold14:2830364-2832333(+)	3	1827+143	609	+
**ABCF Subfamily (3 genes)**						
*AsABCF1*	ASI10006319	scaffold100:2782009-2783929(−)	2	1815+106	605	+
*AsABCF2*	ASI10011705	scaffold5:3835256-3838673(−)	2	2805+613	935	+
*AsABCF3*	ASI10009140	scaffold7:4751204-4753620(+)	4	2175+242	725	+
**ABCG Subfamily (21 genes)**						
*AsABCG2*	ASI10004617	scaffold28:1623903-1626178(−)	5	1899+377	633	+
*AsABCG3*	ASI10008396	scaffold116:2141408-2144367(+)	6	2286+674	762	+
*AsABCG6*	ASI10005115	scaffold77:2634493-2634706(−)	6	1599+331	533	+
*AsABCG7*	ASI10009696	scaffold4:5791835-5801142(−)	7	2091+7217	697	+
*AsABCG10*	ASI10002998	scaffold68:338877-350631(−)	9	2073+9682	691	+
*AsABCG14*	ASI10017482	scaffold14:39864271-39876401(−)	8	1806+10325	602	+
*AsABCG13*	ASI10017484	scaffold14:39904223-39909126(+)	7	2297+2607	869	+
*AsABCG17a*	ASI10002994	scaffold68:308541-310459(−)	3	1779+140	593	+
*AsABCG17b*	ASI10002995	scaffold68:315600-317518(−)	3	1779+140	593	+
*AsABCG18*	ASI10002993	scaffold68:304413-306415(−)	4	1782+241	594	+
*AsABCG19*	ASI10002992	scaffold68:299889-301953(−)	5	1800+265	600	+
*AsABCG20*	ASI10002991	scaffold68:295550-298589(−)	5	1710+1330	570	+
*AsABCG21*	ASI10011562	scaffold5:2012784-2021328(+)	7	1839+6706	613	+
*AsABCG22*	ASI10002996	scaffold68:318211-320277(−)	5	1794+273	598	+
*AsABCG23*	ASI10002997	scaffold68:321662-335687(−)	7	2100+11926	700	+
*AsABCG11*	ASI10003001	scaffold68:361453-367680(−)	8	1995+4233	665	+
*AsABCG24*	ASI10005113	scaffold77:2627164-2629127(−)	6	1584+380	528	-
*AsABCG25*	ASI10005114	scaffold77:2629922-2632124(−)	7	1791+412	597	-
*AsABCG26*	ASI10003266	scaffold6:809083-811434(−)	7	1839+513	613	+
*AsABCG27*	ASI10014141	scaffold16:12792427-12799169(−)	5	1962+4781	654	+
*AsABCG28*	ASI10015886	scaffold14:21804555-21808651(+)	6	1833+17432	611	+
**ABCH Subfamily (3 genes)**						
*AsABCH1*	ASI10006388	scaffold100:3673129-3675938(−)	7	2328+482	776	+
*AsABCH2*	ASI10003817	scaffold91:793497-800786(+)	11	2133+5157	711	+
*AsABCH3*	ASI10017471	scaffold14:39764080-39771407(+)	10	2172+5156	724	+

^a^ “+” indicates the positive strand, and “−” the negative strand. ^b^ “+” indicates that the gene is supported by transcription sequence, and “−” not.

**Table 2:** Gene number comparison of eight ABC transporter subfamilies among genomes of *A. sinensis*, five other insect species, and human being.

ABC Subfamily	*A. sinensis*	*A. gambiae*	*D. melanogaster*	*A. mellifera*	*B. Mori*	*T. castaneum*	*H. sapiens*
**ABCA**	10	9	10	3	6	10	13
**ABCB**	5	5	8	5	8	6	11
**ABCC**	16	13	14	9	15	35	12
**ABCD**	2	2	2	2	2	2	4
**ABCE**	1	1	1	1	1	1	1
**ABCF**	3	3	3	3	3	3	3
**ABCG**	21	16	15	15	13	13	5
**ABCH**	3	3	3	3	3	3	0
**Total**	**61**	**52**	**56**	**41**	**51**	**73**	**48**

**Table 3:** Primer sequences used for qPCR.

Gene Name	Forward Primer (5′–3′)	Reverse Primer (5′–3′)	Size (Base Pairs)
AsABCG28	CAACCTGTACTCCACCACCC	TAACGACCAGACCGAGCAAC	140
AsABCA5	GGGAGTTCAAGTGTCTCGGG	TGTACTGTTTGACGTCCGCT	129
AsABCC9	TGCGTAGCCGGTTGACTATC	AATGAGACAGCTCCAGTGCC	126
AsABCG7	ATCTGAACCTCGGCGGAAAC	CCCAGAACTGGTGGAACTCG	198
AsABCB1	GTAAAACCGGCGAAGTGCTG	GCGTCATGGTGAGAAACACG	177
AsABCB2	GTGAGATCCCGATGCAGGAG	TTCTGACCACCGGACAGTTG	130
AsABCB3	TTCTCGGTTCGGTCATTCGG	ACACAAACTCTACGCCCTCG	150
AsABCB4	GTTTCACGACACCATTCGGC	AACTTAAGGCCACGCTCTCC	156
AsABCB5	ACGGGTTGAACATGGGTGAG	GATAGCACGGGCGATAGCAA	176
AsABCC2	GGAGAGGTGCTGATCGATGG	CTGGGTAGTCCTCGAAGGGA	142
RPS7	CGGAGAAGATGGCATGGGAGAT	ATAGTGAGCATAGGCCCGGTTA	148
RPL49	GGAGCCGGTCGGTGATATGT	TTCCTTCTCGGTCGGCTTCG	121

## Supplementary Materials

The following are available online at https://www.mdpi.com/1422-0067/20/6/1409/s1.
