# Supplementary material for: ATP-Binding Cassette (ABC) Transporter Genes Involved in Pyrethroid Resistance in the Malaria Vector Anopheles sinensis: Genome-Wide Identification, Characteristics, Phylogenetics, and Expression Profile
Source: Int J Mol Sci. 2019 Mar 20;20(6):1409. doi: 10.3390/ijms20061409 (PMC6471920; doi:10.3390/ijms20061409)
Supplement: Supplementary file 1 [file ijms-20-01409-s001.pdf]

Supporting information

ATP-Binding Cassette (ABC) transporter genes involved in pyrethroid resistance in the malaria vector *Anopheles sinensis*: genome-wide identification, characteristics, phylogenetics and expression profile

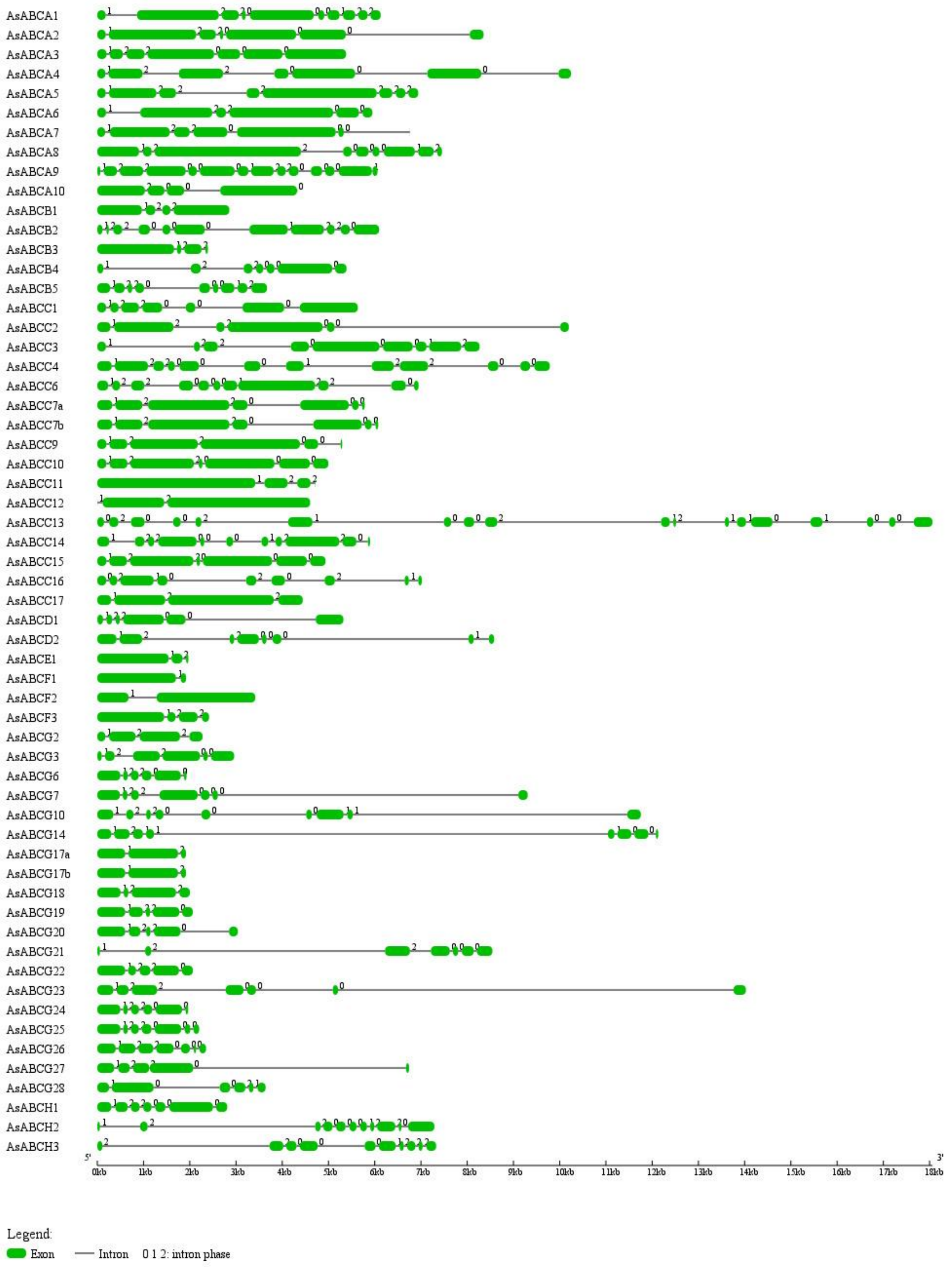

Supplementary Figure 1. Gene structure of predicted *An. sinensis* ABC transporter genes. Green boxes represent exons and black lines represent introns. The numbers indicate the splicing phases of the ABC transporter genes: 0 refers to phase 0; 1 to phase 1; and 2 to phase 2.

(A)

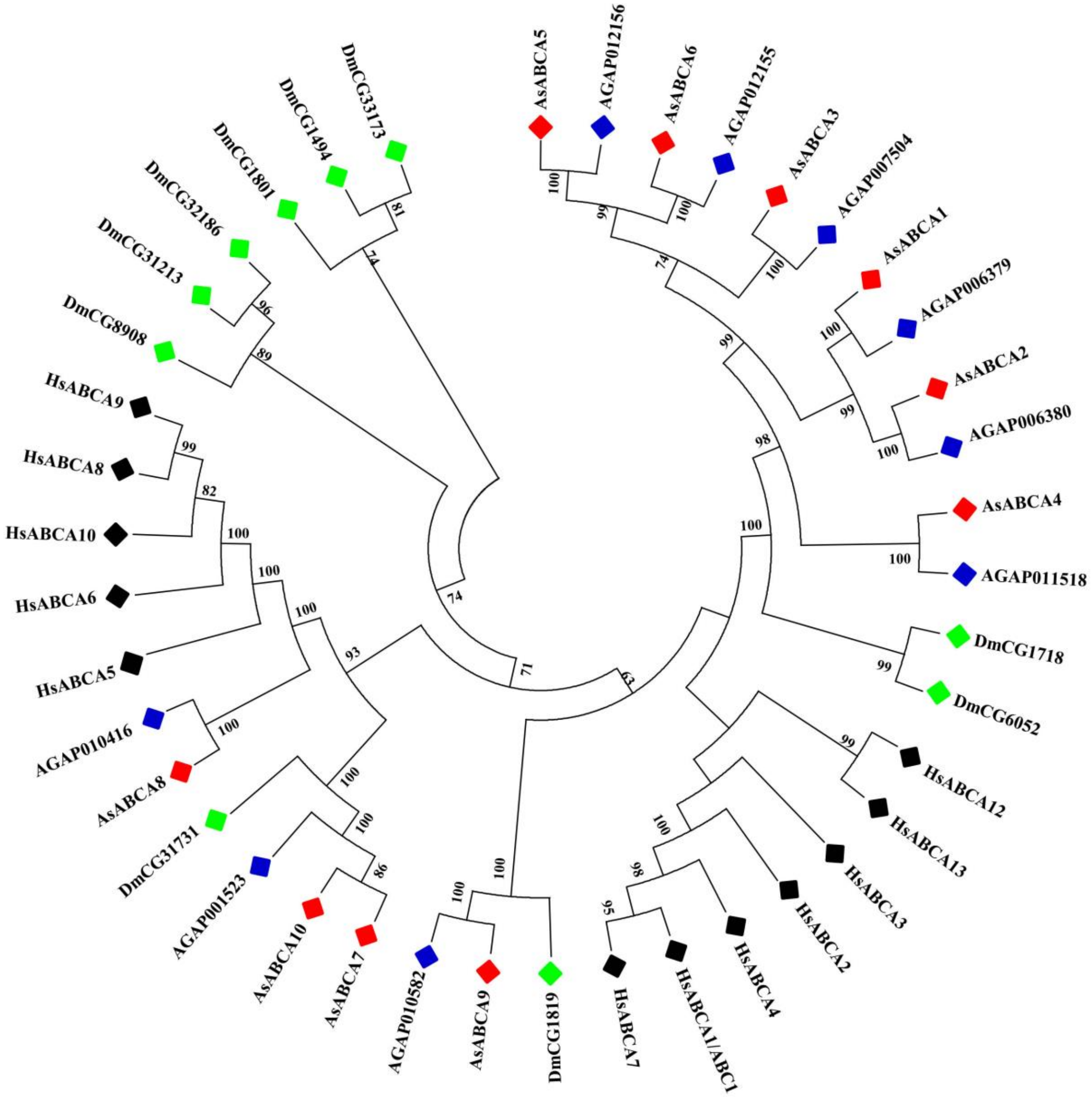
AsABCA5
AGAP012156
AsABCA6
AGAP012155
AsABCA3
AGAP007504
AsABCA1
AGAP006379
AsABCA2
AGAP006380
AsABCA4
AGAP011518
DmCG1718
DmCG6052
HsABCA12
HsABCA13
HsABCA3
HsABCA2
HsABCA4
HsABCA1/ABC1
HsABCA7
DmCG1819
AsABCA9
AGAP010582
AsABCA7
AsABCA10
AGAP001523
DmCG31731
AsABCA8
AGAP010416
HsABCA5
HsABCA6
HsABCA10
HsABCA8
HsABCA9
DmCG8908
DmCG31213
DmCG32186
DmCG1801
DmCG1494
DmCG33173

(B)

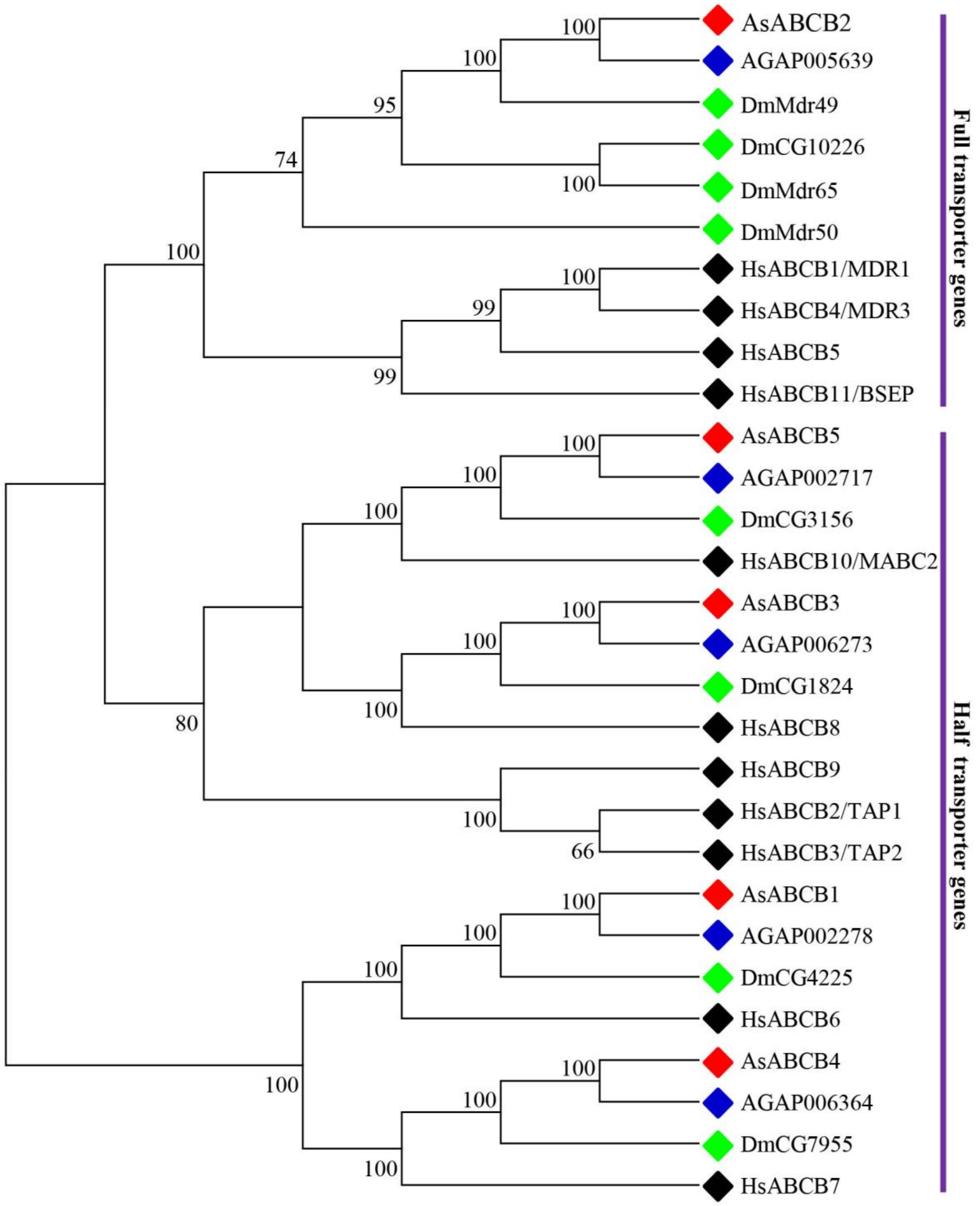

AsABCB2
AGAP005639
DmMdr49
DmCG10226
DmMdr65
DmMdr50
HsABCB1/MDR1
HsABCB4/MDR3
HsABCB5
HsABCB11/BSEP
AsABCB5
AGAP002717
DmCG3156
HsABCB10/MABC2
AsABCB3
AGAP006273
DmCG1824
HsABCB8
HsABCB9
HsABCB2/TAP1
HsABCB3/TAP2
AsABCB1
AGAP002278
DmCG4225
HsABCB6
AsABCB4
AGAP006364
DmCG7955
HsABCB7
Full transporter genes
Half transporter genes

(C)

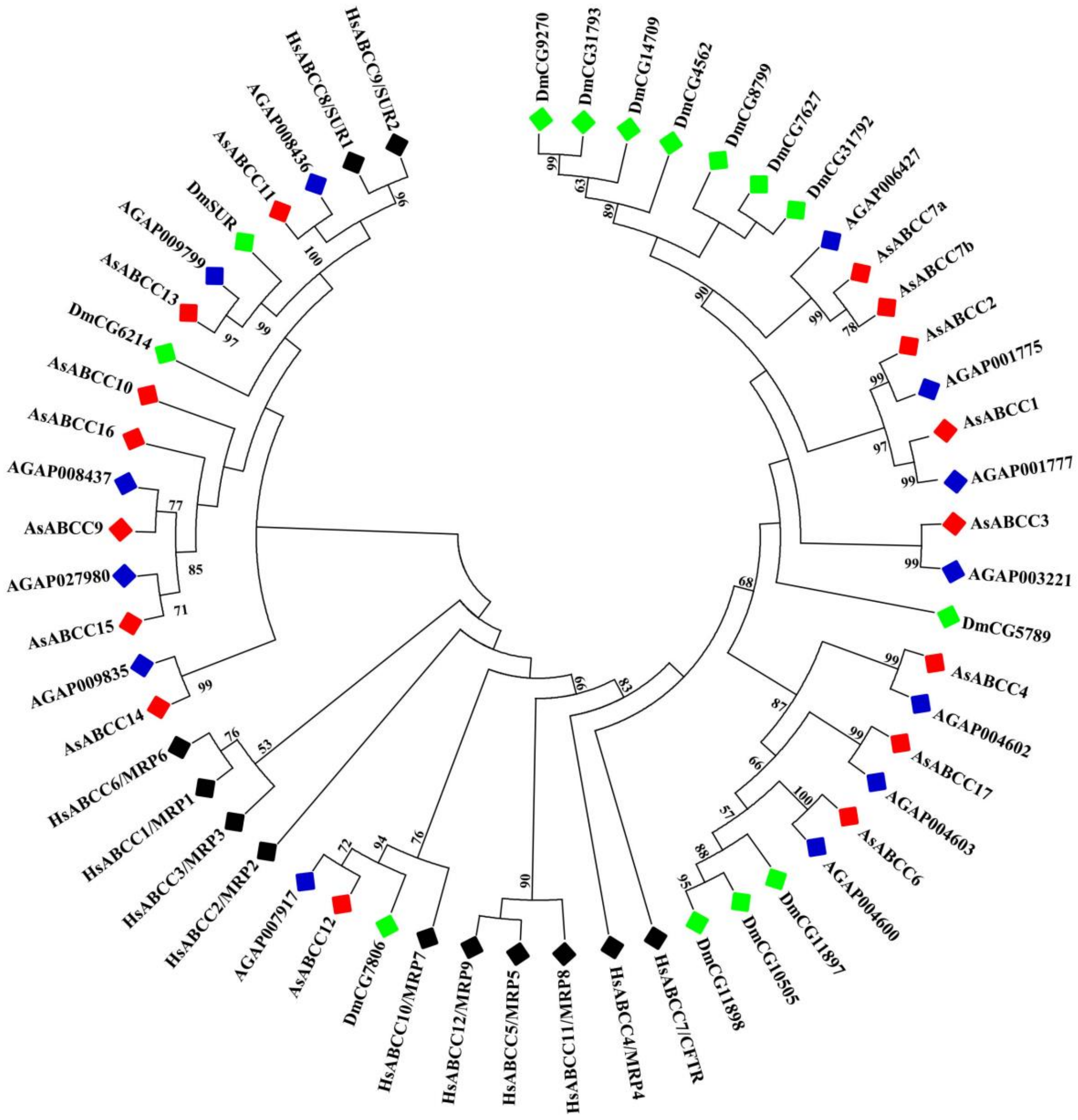

HsABCC9/SUR2
HsABCC8/SUR1
AGAP008436
AsABCC11
DmSUR
AGAP009799
AsABCC13
DmCG6214
AsABCC10
AsABCC16
AGAP008437
AsABCC9
AGAP027980
AsABCC15
AGAP009835
AsABCC14
HsABCC6/MRP6
HsABCC1/MRP1
HsABCC3/MRP3
HsABCC2/MRP2
AGAP007917
AsABCC12
DmCG7806
HsABCC10/MRP7
HsABCC12/MRP9
HsABCC5/MRP5
HsABCC11/MRP8
HsABCC4/MRP4
HsABCC7/CFTR
DmCG11898
DmCG10505
DmCG11897
AGAP004600
AsABCC6
AGAP004603
AsABCC17
AGAP004602
AsABCC4
DmCG5789
AGAP003221
AsABCC3
AGAP001777
AsABCC1
AGAP001775
AsABCC2
AsABCC7b
AsABCC7a
AGAP006427
DmCG31792
DmCG7627
DmCG8799
DmCG4562
DmCG14709
DmCG31793
DmCG9270

(D)

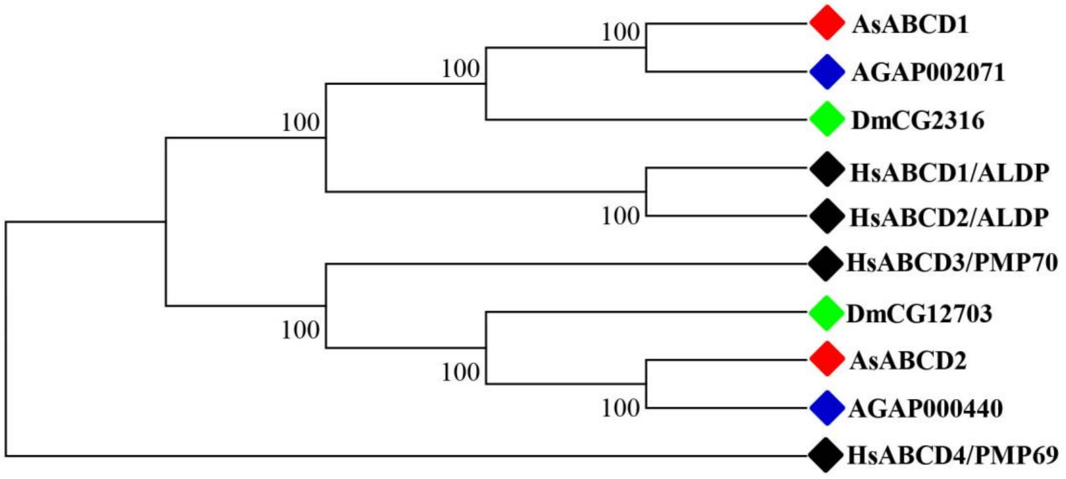
AsABCD1
AGAP002071
DmCG2316
HsABCD1/ALDP
HsABCD2/ALDP
HsABCD3/PMP70
DmCG12703
AsABCD2
AGAP000440
HsABCD4/PMP69
100
100
100
100
100
100
100
100

(E)

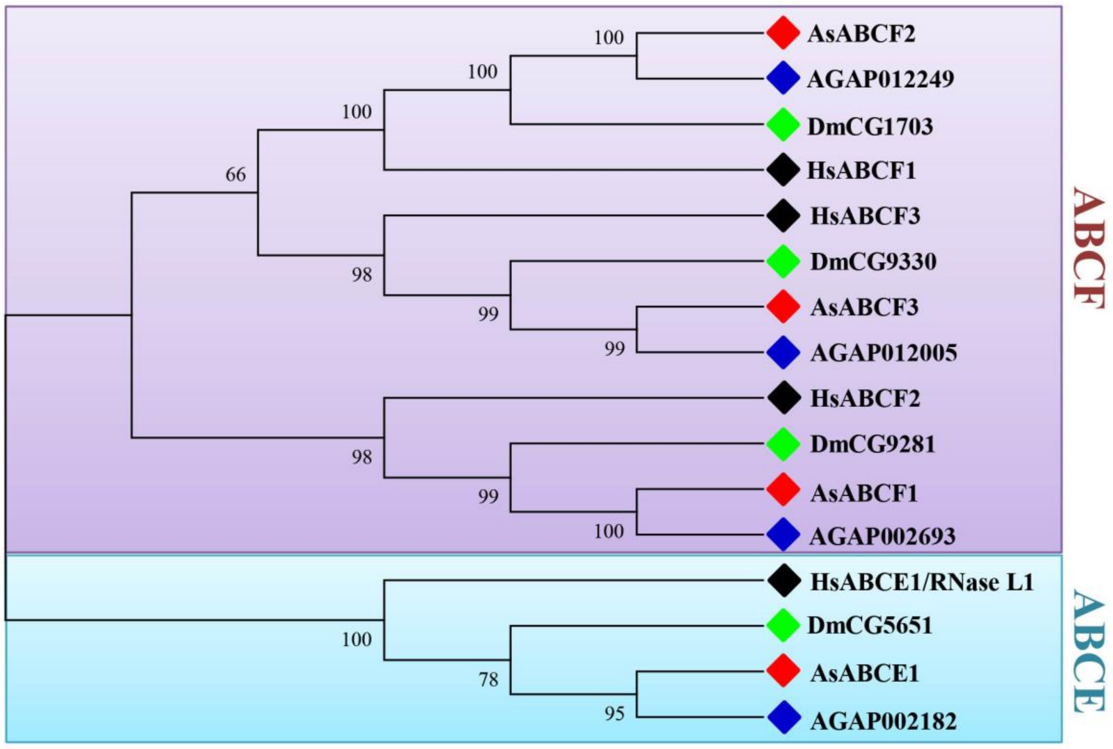
AsABCF2
AGAP012249
DmCG1703
HsABCF1
HsABCF3
DmCG9330
AsABCF3
AGAP012005
HsABCF2
DmCG9281
AsABCF1
AGAP002693
HsABCE1/RNase L1
DmCG5651
AsABCE1
AGAP002182
ABCF
ABCE
100
100
100
66
98
99
99
98
99
100
100
78
95

(F)

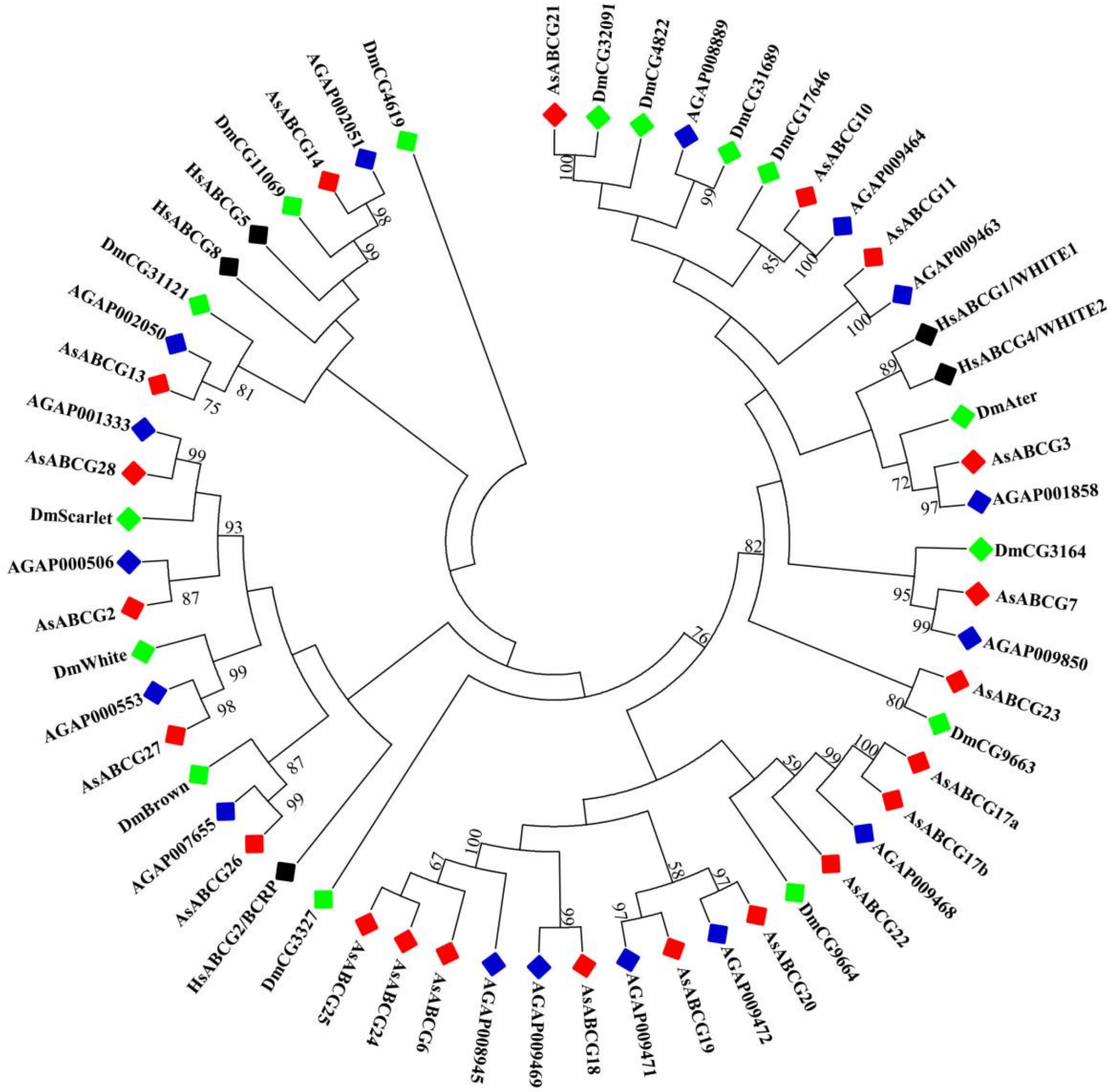
AsABCG21
DmCG32091
DmCG4822
AGAP008889
DmCG31689
DmCG17646
AsABCG10
AGAP009464
AsABCG11
AGAP009463
HsABCG1/WHITE1
HsABCG4/WHITE2
DmAter
AsABCG3
AGAP001858
DmCG3164
AsABCG7
AGAP009850
AsABCG23
DmCG9663
AsABCG17a
AsABCG17b
AGAP009468
AsABCG22
DmCG9664
AsABCG20
AGAP009472
AsABCG19
AGAP009471
AsABCG18
AGAP009469
AGAP008945
AsABCG6
AsABCG24
AsABCG25
DmCG3327
HsABCG2/BCRP
AsABCG26
AGAP007655
DmBrown
AsABCG27
AGAP000553
DmWhite
AsABCG2
AGAP000506
DmScarlet
AsABCG28
AGAP001333
AsABCG13
AGAP002050
DmCG31121
HsABCG8
HsABCG5
DmCG11069
AsABCG14
AGAP002051
DmCG4619

(G)

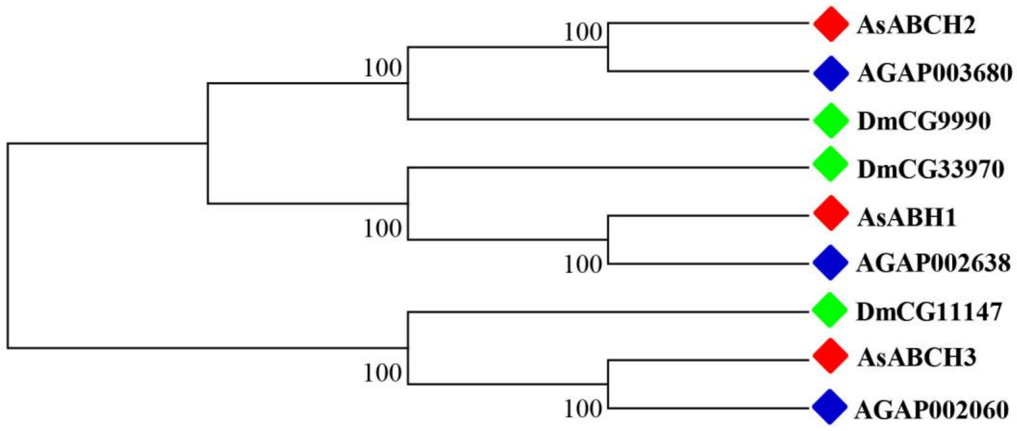

Supplementary Figure 2. Phylogenetic relationships of subfamilies of ABC transporter genes of *An. sinensis*, *An. gambiae*, *Dr. melanogaster* and *Ho. sapiens*. (A) ABCA subfamily, (B) ABCB subfamily, (C) ABCC subfamily, (D) ABCD subfamily, (E) ABCE and ABCF subfamilies, (F) ABCG subfamily, and (G) ABCH subfamily. These trees were constructed using the maximum-likelihood method based on predicted amino acid sequences. The program package MEGA5.0 was used for the construction, and the gaps of alignments were deleted with pairwise deletion method. Bootstraps values resulting from 1000 replications are marched on the branches. As, *An. sinensis* (in red); AG, *An. gambiae* (in blue); Dm, *Dr. melanogaster* (in green); Hs, *Ho. sapiens* (in black).

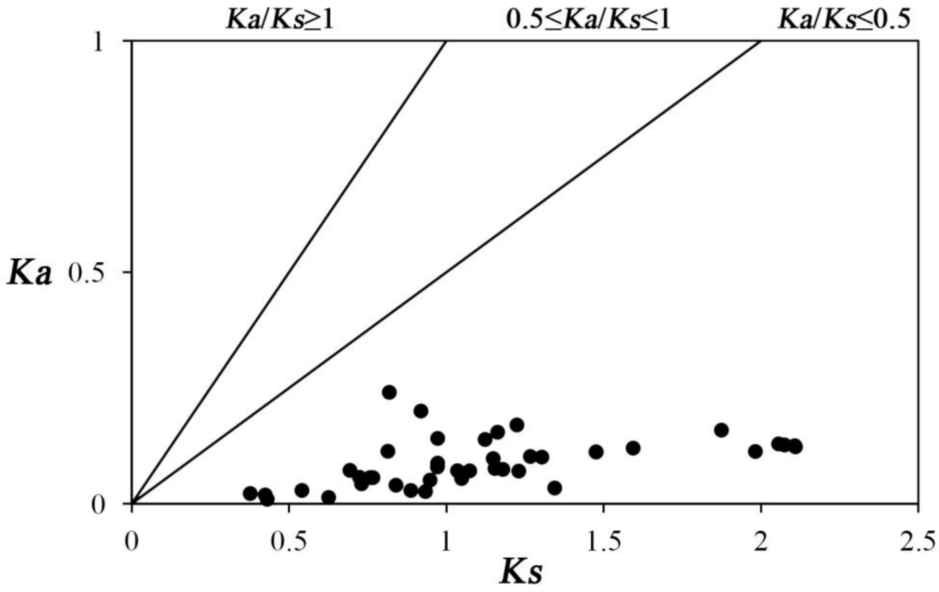

Supplementary Figure 3. *Ka*/*Ks* distribution for ABC transporter orthologous genes. The *Ka/Ks* ratios of all genes are less than 0.5. These data indicate that all genes experienced purifying selection.

Supplementary Table S1. RNA-Seq results of ABC transporter genes of *An. sinensis* in all three field pyrethroid-resistant populations (AH-FR，CQ-FR and YN-FR) in comparison with laboratory susceptible strain (WX-LS). The significantly differentially expressed genes are shown in bold.

Gene ID	FPKM of DGE RNA-seq sample				AH-FR vs WX-LS		CQ-FR vs WX-LS		YN-FR vs WX-LS	
	AH-FR	CQ-FR	YN-FR	WX-LS	FPKM log2(fold)	*P*-value	FPKM log2(fold)	*P*-value	FPKM log2(fold)	*P*-value
ASI10006665	13.20415	7.185765	9.123355	16.778167	-0.304434235	0.6754	-1.31028202	0.0526	-0.772321648	0.37425
ASI10006666	44.73765	14.8831	33.4433	33.081867	0.476617138	0.52645	-1.239267261	0.0621	0.122274036	0.896
ASI10001787	1.739285	1.21528	1.51622	1.4437733	0.309798195	1	-0.335452497	1	0.177278398	1
ASI10005446	0	0.1220655	0.309123	0.0645504	-0.092225979	1	0.832242537	1	2.3663533	1
**ASI10011585**	**13.9094**	**1.76993**	**5.895405**	**4.9853833**	**1.521454229**	**0.0435**	**-1.580904636**	**0.0463**	**0.34850563**	**0.67125**
ASI10011586	0.812307	0.296256	0.303761	0.345195	1.275770366	1	-0.307455997	1	-0.077863312	1
ASI10017076	6.25515	10.3745	3.65677	4.9432667	0.380678241	0.59335	0.982606253	0.1604	-0.328272305	0.69875
ASI10008148	0.5634185	0.1771875	0.2265925	0.222874	1.379125478	1	-0.417847742	1	0.130495354	1
ASI10012001	10.65735	5.69763	12.82535	11.113467	-0.019321926	0.97885	-1.050775396	0.116	0.313314667	0.71985
ASI10008529	0	0	0.5619525	0.7448417	-0.816984647	1	-0.826506591	1	-0.299852105	1
ASI10016633	25.97025	19.0789	27.86545	28.1755	-0.0764851	0.9363	-0.649360618	0.4451	0.090667176	0.93945
ASI10010010	3.177745	2.252	2.95841	3.2148533	0.024405805	1	-0.600462114	1	-0.013322797	1
ASI10009929	2.574345	4.2492	5.594355	4.16732	-0.653760627	0.4599	-0.058810615	0.94695	0.53147492	0.54205
ASI10006641	14.2093	20.0868	19.07315	15.812267	-0.113054761	0.8756	0.258309403	0.6925	0.377116287	0.64825
ASI10016463	5.415525	6.79192	10.9268	7.2339733	-0.376567081	0.6274	-0.177872956	0.8189	0.701663518	0.3914
ASI10014633	4.564405	5.228445	6.684985	6.5224467	-0.473870629	0.5151	-0.405932394	0.56695	0.14213707	0.85725
ASI10014632	14.1126	9.068125	13.78205	10.27273	0.499341551	0.4866	-0.266839609	0.6852	0.530585237	0.53395
ASI10014742	3.49632	4.13408	8.167355	4.2739267	-0.24852529	0.72665	-0.134900194	0.8534	1.040924193	0.20725
ASI10004082	9.27102	4.61573	15.84405	6.8807	0.471300341	0.5095	-0.662901068	0.338	1.309945926	0.12835
ASI10004081	0.245234	0.3367445	0.428552	0.3335867	-0.402787483	1	-0.07330209	1	0.468021761	1
ASI10001723	1.20499	2.246755	2.272165	1.92673	-0.635989886	1	0.134754051	1	0.344619264	1
ASI10002723	1.668935	2.85607	2.36831	1.76999	-0.043660806	1	0.603360108	1	0.526810078	1
**ASI10007114**	**6.782115**	**1.629245**	**3.694845**	**2.2926967**	**1.605884815**	**0.0412**	**-0.57974186**	**1**	**0.79507719**	**0.34565**
ASI10007112	5.782505	3.365245	5.07833	4.9585967	0.262921549	0.71235	-0.646102104	0.37385	0.141022345	0.8616
**ASI10007115**	**17.675**	**11.8014**	**18.11215**	**35.0428**	**-0.946275089**	**0.202**	**-1.657033597**	**0.01455**	**-0.845542937**	**0.35055**
ASI10003245	8.84066	8.64466	15.3278	7.9454167	0.195154589	0.78205	0.034789775	0.9576	1.054593734	0.22035
ASI10001014	0.672789	0.297085	0.698664	1.19318	-0.785408448	1	-2.092764292	1	-0.665507524	1
ASI10009681	8.83829	13.98345	13.74465	7.63816	0.25171902	0.7197	0.785529523	0.24555	0.954175937	0.24835
ASI10007113	3.938775	0.600704	2.73477	2.5773133	0.653061131	0.39135	-2.188010726	1	0.192130106	1
ASI10009679	13.5209	20.6	17.06365	12.064067	0.205613368	0.7779	0.685041685	0.32855	0.606852958	0.4563
ASI10004083	2.81675	3.2304	3.079055	2.4246667	0.257357306	1	0.327018069	0.6736	0.451357026	0.59665
ASI10017457	16.88395	11.70595	20.6265	20.5218	-0.240314587	0.73985	-0.896824095	0.18375	0.113993527	0.8919
ASI10013565	23.77695	34.0781	37.9187	38.280133	-0.645889332	0.39005	-0.254682166	0.71795	0.093007554	0.9201
ASI10014410	233.119	244.553	297.687	194.81967	0.300115932	0.67915	0.241071737	0.69515	0.718333406	0.46795
ASI10006319	203.511	220.1315	223.3655	223.232	-0.092232097	0.9006	-0.107114392	0.86415	0.107532533	0.9157
ASI10011705	29.5323	36.8468	34.4426	38.4155	-0.338140397	0.64885	-0.147099758	0.81915	-0.0508502	0.9578
ASI10009140	59.53895	60.94865	48.3878	45.2049	0.438557501	0.5597	0.344185092	0.59855	0.204839635	0.82805
ASI10004617	0.805827	1.738645	3.86451	0.568342	0.544878978	1	1.526272939	1	2.872029312	0.1191

ASI10008396	1.358485	2.01803	1.41983	0.8371747	0.739554983	1	1.182463772	1	0.868722521	1
ASI10005115	6.096745	5.895285	10.8691	6.05948	0.049984806	0.9561	-0.126518284	0.8792	0.949542703	0.2828
**ASI10009696**	**66.42685**	**24.79995**	**66.78665**	**24.1165**	**1.502935216**	**0.04395**	**-0.046587828**	**0.94175**	**1.576165527**	**0.08275**
ASI10002998	9.039075	5.13065	7.09952	11.955	-0.362173138	0.623	-1.307317478	0.1016	-0.645188126	0.43765
ASI10017482	7.87822	10.329	6.28131	10.21685	-0.33382944	0.65575	-0.071138185	0.9158	-0.595184312	0.50825
ASI10017484	0.5259825	0.4903005	1.36251	0.53955	0.004422461	1	-0.224986291	1	1.443056735	1
ASI10002994	1.0138205	1.119735	1.4361	2.6098267	-1.322969044	0.28205	-1.307972215	0.30135	-0.755233012	1
ASI10002995	0	0.109338	0.2583355	0.1015158	-0.142516694	1	0.021924681	1	1.454265985	1
ASI10002993	18.92305	15.36225	21.75775	25.789867	-0.405521731	0.5698	-0.834285934	0.2215	-0.138694247	0.86575
ASI10002992	2.67197	0.3742235	2.950265	0.5678643	2.275433687	1	-0.688494053	1	2.483799182	1
ASI10002991	23.28805	17.23195	19.81475	16.811633	0.511291793	0.4704	-0.051290945	0.93795	0.343795706	0.6662
ASI10011562	5.64435	3.356585	6.22002	5.37822	0.110839038	0.90455	-0.767023599	0.43035	0.316402507	0.75685
ASI10002996	0	0.371788	0.1790385	0.1912427	-0.257699808	1	0.872215054	1	0.011442146	1
**ASI10002997**	**17.21105**	**6.93384**	**19.40685**	**18.721633**	**-0.080217499**	**0.906**	**-1.519864962**	**0.0403**	**0.158484706**	**0.84875**
ASI10003001	38.97295	18.4467	31.4412	35.1735	0.189123325	0.79305	-1.018034494	0.12675	-0.055197602	0.95075
ASI10005113	0.4909205	2.171975	1.797525	1.4500867	-1.521445454	1	0.496021258	1	0.416437962	1
ASI10005114	0.829031	1.441	1.078395	0.398728	1.097177091	1	1.766713151	1	1.541951183	1
ASI10003266	62.2466	24.5125	57.45905	50.275767	0.349259123	0.63615	-1.123233086	0.0887	0.299315214	0.74135
ASI10014141	4.159905	10.051835	9.74226	4.3467667	-0.022223043	0.94875	1.122510935	0.24445	1.270998919	0.23105
**ASI10015886**	**13.7664**	**19.62915**	**25.34355**	**5.1081633**	**1.471400339**	**0.04955**	**1.855211475**	**0.0092**	**2.417370711**	**0.00775**
ASI10006388	7.711095	2.918995	9.22602	5.5424767	0.517610882	0.47045	-1.011939717	0.19915	0.841775598	0.2983
ASI10003817	6.10794	1.0032175	4.065725	2.69066	1.223897896	0.1586	-1.510233854	1	0.702189007	0.4627
ASI10017471	0.3670665	0.244019	0.507556	1.3428	-1.829937261	1	-2.547087646	1	-1.296979253	1

Supplementary Table 2. Accession numbers of ABC transporter genes from *An. gambiae*, *Dr. melanogaster* and *Ho. sapiens*

**Genes**	**Accession no.**	**Genes**	**Accession no.**	**Genes**	**Accession no.**
AGAP000440	XP_310656.2	DmAter	NP_523471.2	HsABCA1/ABC1	NP 005493.2
AGAP000506	XP_310585.4	DmBrown	NP_523824.1	HsABCA10	NP_525021.3
AGAP000553	XP_310530.3	DmCG10226	NP_648040.1	HsABCA12	NP_056472.2
AGAP001333	XP_321812.4	DmCG10505	NP_611571.2	HsABCA13	NP_689914.3
AGAP001523	XP_321601.5	DmCG11069	NP_651307.2	HsABCA2	NP_001597.2
AGAP001775	XP_551022.4	DmCG11147	NP 608954.1	HsABCA3	NP_001080.2
AGAP001777	XP_321300.4	DmCG11897	NP_651678.1	HsABCA4	AAC05632.1
AGAP001858	XP_321211.4	DmCG11898	NP_651679.2	HsABCA5	NP_061142.2
AGAP002050	XP_320998.5	DmCG12703	NP_608354.1	HsABCA6	NP_525023.2
AGAP002051	XP_320996.5	DmCG14709	NP_650086.2	HsABCA7	AAK00959.1
AGAP002060	XP-320987.5	DmCG1494	NP_608444.2	HsABCA8	NP_009099.1
AGAP002071	XP_320975.4	DmCG1703	NP_572736.1	HsABCA9	NP_525022.2
AGAP002182	XP_308004.3	DmCG1718	NP_608445.2	HsABCB1/MDR1	NP_001335874.1
AGAP002278	XP_307900.5	DmCG17646	NP_608618.1	HsABCB10/MABC2	NP_036221.2
AGAP002638	XP 312290.4	DmCG1801	NP_728408.3	HsABCB11/BSEP	XP_016860654.1
AGAP002693	XP_312228.5	DmCG1819	NP_001138224.1	HsABCB2/TAP1	NP_000584.2
AGAP002717	XP_312209.4	DmCG1824	NP_572810.1	HsABCB3/TAP2	AAA59841.1
AGAP003221	XP_312930.5	DmCG2316	NP_651906.1	HsABCB4/MDR3	NP_000434.1
AGAP003680	XP 313462.4	DmCG31121	NP_733058.1	HsABCB5	AAP55848.1
AGAP004600	XP_001237640.2	DmCG31213	NP_001262752.1	HsABCB6	NP_005680.1
AGAP004602	XP_001688623.2	DmCG3156	NP_569844.2	HsABCB7	NP_001258625.1
AGAP004603	XP_315221.5	DmCG3164	NP_608494.2	HsABCB8	NP_001269220.1
AGAP005639	XP_315658.3	DmCG31689	NP_722827.1	HsABCB9	NP_062571.1
AGAP006273	XP_316337.4	DmCG31731	NP_001036360.1	HsABCC1/MRP1	AAB46616.1
AGAP006364	XP_316391.4	DmCG31792	NP_724148.2	HsABCC10/MRP7	NP_258261.2
AGAP006379	XP_557048.3	DmCG31793	NP_609930.4	HsABCC11/MRP8	NP_149163.2
AGAP006380	XP_001688862.1	DmCG32091	NP_729728.1	HsABCC12/MRP9	NP_150229.2
AGAP006427	XP_316463.3	DmCG32186	NP_730301.3	HsABCC2/MRP2	AAB39892.1
AGAP007504	XP_308371.4	DmCG33173	NP_788912.2	HsABCC3/MRP3	NP_003777.2
AGAP007655	XP_308215.4	DmCG3327	NP_995622.1	HsABCC4/MRP4	NP_005836.2
AGAP007917	XP_317569.4	DmCG33970	NP 01034071.1	HsABCC5/MRP5	AAD37716.1
AGAP008436	XP_317007.3	DmCG4225	NP_650503.1	HsABCC6/MRP6	O95255.2
AGAP008437	KFB39079.1	DmCG4562	NP_001262747.1	HsABCC7/CFTR	AAC13657.1
AGAP008889	XP_319635.4	DmCG4619	NP_609329.3	HsABCC8/SUR1	AAB02278.1
AGAP008945	KFB35656.1	DmCG4822	NP_722602.1	HsABCC9/SUR2	NP_005682.2
AGAP009463	XP_310233.4	DmCG5651	NP_648272.1	HsABCD1/ALDP	CAA79922.1
AGAP009464	XP_310232.4	DmCG5789	NP_651269.1	HsABCD2/ALDP	NP_005155.1
AGAP009468	XP_001688274.1	DmCG6052	NP_649002.2	HsABCD3/PMP70	CAA41416.1
AGAP009469	XP_001688273.1	DmCG6214	NP_723772.2	HsABCD4/PMP69	NP_005041.1
AGAP009471	XP_310228.4	DmCG7627	NP_609215.3	HsABCE1/RNase L1	CAA53972.1
AGAP009472	XP_001688272.1	DmCG7806	NP_609207.1	HsABCF1	AAH34488.1

AGAP009799	XP_553659.3	DmCG7955	NP_728642.2	HsABCF2	NP_005683.2
AGAP009835	XP_553715.3	DmCG8799	NP_610482.3	HsABCF3	NP_060828.2
AGAP009850	XP_318963.3	DmCG8908	NP_611464.3	HsABCG1/WHITE1	NP_004906.3
AGAP010416	XP_311532.4	DmCG9270	AFI71925.1	HsABCG2/BCRP	NP_004818.2
AGAP010582	XP_314549.4	DmCG9281	NP_573057.1	HsABCG4/WHITE2	NP_071452.2
AGAP011518	ETN65103.1	DmCG9330	NP_649129.1	HsABCG5	NP_071881.1
AGAP012005	XP_320530.4	DmCG9663	NP_608760.2	HsABCG8	AAI13658.1
AGAP012155	XP_320377.4	DmCG9664	NP_722889.1		
AGAP012156	XP_552044.3	DmCG9990	NP 651628.1		
AGAP012249	XP_320293.4	DmMdr49	NP_523724.2		
AGAP027980	ETN62869.1	DmMdr50	NP_523740.3		
		DmMdr65	NP_476831.1		
		DmScarlet	NP_524108.1		
		DmSUR	Q9VL32.4		
		DmWhite	NP_476787.1		
